# Structural basis for breadth development in the HIV-1 V3-glycan targeting DH270 antibody clonal lineage

**DOI:** 10.1038/s41467-023-38108-1

**Published:** 2023-05-15

**Authors:** Rory Henderson, Ye Zhou, Victoria Stalls, Kevin Wiehe, Kevin O. Saunders, Kshitij Wagh, Kara Anasti, Maggie Barr, Robert Parks, S. Munir Alam, Bette Korber, Barton F. Haynes, Alberto Bartesaghi, Priyamvada Acharya

**Affiliations:** 1 Department of Medicine and Immunology, Duke University School of Medicine, Durham, NC USA; 2 Duke Human Vaccine Institute, Duke University School of Medicine, Durham, NC USA; 3 Department of Computer Science, Duke University, Durham, NC USA; 4 Department of Surgery, Duke University School of Medicine, Durham, NC USA; 5 Theoretical Biology and Biophysics, Los Alamos National Laboratory, Los Alamos, NM USA; 6 New Mexico Consortium, Los Alamos, NM USA; 7 Department of Pathology, Duke University School of Medicine, Durham, NC USA; 8 Department of Biochemistry, Duke University School of Medicine, Durham, NC USA; 9 Department of Electrical and Computer Engineering, Duke University, Durham, NC USA

**Keywords:** Cryoelectron microscopy, Vaccines, Retrovirus

## Abstract

Antibody affinity maturation enables adaptive immune responses to a wide range of pathogens. In some individuals broadly neutralizing antibodies develop to recognize rapidly mutating pathogens with extensive sequence diversity. Vaccine design for pathogens such as HIV-1 and influenza has therefore focused on recapitulating the natural affinity maturation process. Here, we determine structures of antibodies in complex with HIV-1 Envelope for all observed members and ancestral states of the broadly neutralizing HIV-1 V3-glycan targeting DH270 antibody clonal B cell lineage. These structures track the development of neutralization breadth from the unmutated common ancestor and define affinity maturation at high spatial resolution. By elucidating contacts mediated by key mutations at different stages of antibody development we identified sites on the epitope-paratope interface that are the focus of affinity optimization. Thus, our results identify bottlenecks on the path to natural affinity maturation and reveal solutions for these that will inform immunogen design aimed at eliciting a broadly neutralizing immune response by vaccination.

## Abstract (web summary)

In this study, Henderson and Zhou et al. visualize the development of a HIV-1 broadly neutralizing antibody (bnAb) from germline to maturity by determining cryo-EM structures of HIV-1 Envelope (Env) proteins bound to Fab fragments of antibodies at different stages of development of a Env V3-glcyan supersite targeting bnAb clone.

## Introduction

Antibody affinity maturation from germline-encoded heavy and light chain immunoglobulin (Ig) genes involves iterative rounds of somatic hypermutation and selection followed by B cell expansion and differentiation, ultimately leading to pathogen neutralization^1^. The advent of high-throughput next-generation sequencing and methods for tracing antibody development has allowed close monitoring of the affinity maturation process^2,3^. Extensive structure-based studies of antibodies alone or of antibodies in contact with cognate antigen have revealed that affinity gains involve acquisition of mutations that improve antibody-antigen contacts, shape complementarity, paratope rigidity, and antibody conformation^2,4–8^. Maturation also involves affinity-independent diversification, which, together, lead to a broad range of potential solutions to the affinity optimization problem^4,9^. Antibodies with neutralization breadth can effectively bind to target antigen despite sequence variability and are a major vaccine design target for pathogens such as HIV-1, influenza, and SARS-CoV-2 and other human coronaviruses^10–12^.

While influenza and SARS-infections are usually cleared within a relatively short time after infection, HIV-1 is a chronic, genome-integrating infection and antibody maturation resulting in broadly neutralizing antibodies (bnAbs) only occurs over multiple years. Defining antibody maturation pathways to strategically inform immunogen design and enable acceleration of this process through vaccination is a primary goal of current HIV-1 vaccine efforts^13^. Broadly neutralizing antibodies have been isolated from people living with HIV (PLWH) and provide the basis for many vaccine strategies aiming to induce bnAbs^14^. Development of HIV-1-directed bnAbs typically requires a defined set of specifically positioned mutations in heavy/light chain Ig pairs to occur in the antibody clone, presenting a formidable challenge in immunogen design aimed at recapitulating bnAb development. This is exacerbated by HIV-1 bnAb features that are uncommon including extensive somatic mutations, long heavy chain third complementarity determining regions (HCDR3s), Ig insertions and deletions, restricted germline gene usage, and enrichment for low probability mutations^15–17^. A detailed understanding of the affinity maturation steps leading to bnAb development, including discrimination among acquired mutations that lead to the desired bnAb response versus those that culminate in less productive off-target responses, is therefore essential to identify the determinants of affinity matured bnAb B cell receptor (BCR) selection.

Studying the coevolution of virus and antibody clones during HIV infection informs vaccine design by defining the HIV-1 Envelope (Env) variants that evolve during bnAb development, thus providing a blueprint for iterative immunogen design^7,18–20^. The targets for HIV-1 bnAbs are conserved epitopes on the Env protein^21–24^. The HIV-1 Env is a trimer of gp120-gp41 heterodimers that are heavily shielded from the host immune systems by N-linked glycosylation and further protected from neutralization by conformational masking and considerable sequence variability.

A glycosylated region near the base of the third variable loop (V3) of HIV-1 Env forms a supersite of vulnerability that is targeted by antibodies originating from diverse germline genes in multiple HIV-1 infected individuals^25^. The development of a broadly neutralizing V3-glycan antibody was studied in an African male living with AIDS from Malawi (CH848, clade C), who was followed from the time of infection up to 5 years after transmission^20^. In the CH848 individual, the early appearance of two autologous “cooperating” neutralizing B cell clones (DH272 and DH475) led to the selection of viral escape variants, that in turn stimulated the DH270 clone that further developed to acquire potent neutralization breadth^20^. The DH270 lineage is derived from the VH1-2*02 and Vλ2-23 heavy and light chain genes, respectively, with a heavy chain complementary determining region length of twenty amino acid residues^20^, DH270.6, the lineage member with the greatest breadth and potency, neutralizes fifty-one percent of a multiclade pseudovirus panel with a geometric mean potency of ~0.21 µg/ml^26^. With somatic mutation levels of 12.8 and 6.7 percent for the heavy and light chain genes, respectively, DH270.6 is among the least mutated V3-glycan bnAbs^26^. DH270 antibodies were detected in the CH848 individual at week 186 and coincided with the appearance of Env variants with a shortened variable loop 1 (V1)^20^. The inferred DH270 unmutated common ancestor antibody (DH270.UCA), representing the naïve B cell progenitor of the DH270 clone, does not neutralize heterologous HIV-1, although a single amino acid change at position 57 of the heavy chain that substituted a glycine for an arginine (G57R) resulted in heterologous HIV-1 neutralization, albeit with limited breadth. As the DH270 clone affinity matured and antibodies accumulated mutations in their heavy and light chain variable regions (V_H_ and V_L_), breadth and potency of heterologous neutralization improved. The early DH270 ancestral intermediate antibodies were able to neutralize heterologous viruses with short V1 loops. The DH270 B cell clonal lineage evolved to gain capacity to neutralize viruses with longer V1 loops, although with reduced potency^20^. A similar inverse correlation between potency and V1 length was observed for other V3-glycan bnAbs 10-1074, PGT121, and PGT128^27,28^. The rich dataset of co-evolving antibodies and Envs available for the DH270 clone^20^ presented an opportunity to study structural aspects of affinity maturation and virus coevolution at an unprecedented level of detail and to define how this information will inform vaccine design aimed at eliciting bnAbs targeting the V3-glycan supersite.

Three key discoveries have informed our current understanding of the DH270 clone. First, though somatic hypermutation is a stochastic process, hot spots and cold spots exist in the antibody sequence for activation-induced cytidine deaminase (AID)- mediated somatic mutations, leading to differences in the probabilities with which antibody residues are mutated. This, combined with the possible need for multiple base changes, can lead to certain mutations occurring with low probability, defined here as less than two percent. HIV-1 bnAbs, including those in the DH270 clone, are enriched for key, low probability mutations^17^. Second, of the forty-two mutations in the most broad and potent member in this clone, only twelve are needed to reach ninety percent of its breadth, allowing us to pinpoint the most critical regions of the antibody that need to be optimized^29^. Finally, key for considering affinity maturation in the context of vaccine development, we have shown that rationally designed immunogens can expand DH270 bnAb V3-glycan precursors^21^.

Here we used cryo-EM to visualize the interactions of a complete inferred DH270 clonal tree with the co-evolving virus Env. We define the defenses mounted by the virus to shield its V3-glycan epitope during infection and demonstrate how the DH270 clone developed to first engage Env and then matured to effectively circumvent these barriers to achieve neutralization breadth. Our results show that clone development involved sequential solutions to affinity gain at specific antibody-antigen contact sites. At each stage of development, the acquisition of a solution was followed by splitting of the clone into distinct sub-clones leading to differential gains in affinity and neutralization breadth. These results showed that mutations acquired early in the clone determined the fate of downstream DH270 clonal affinity maturation. Antibody maturation involved staged, site specific optimization of epitope-paratope contacts with the site of focus shifting as evasive mutations accumulated on the antigenic surface.

## Results

### Structural determination of the Env bound DH270 clone antibody Fabs

**Fig. 1:**
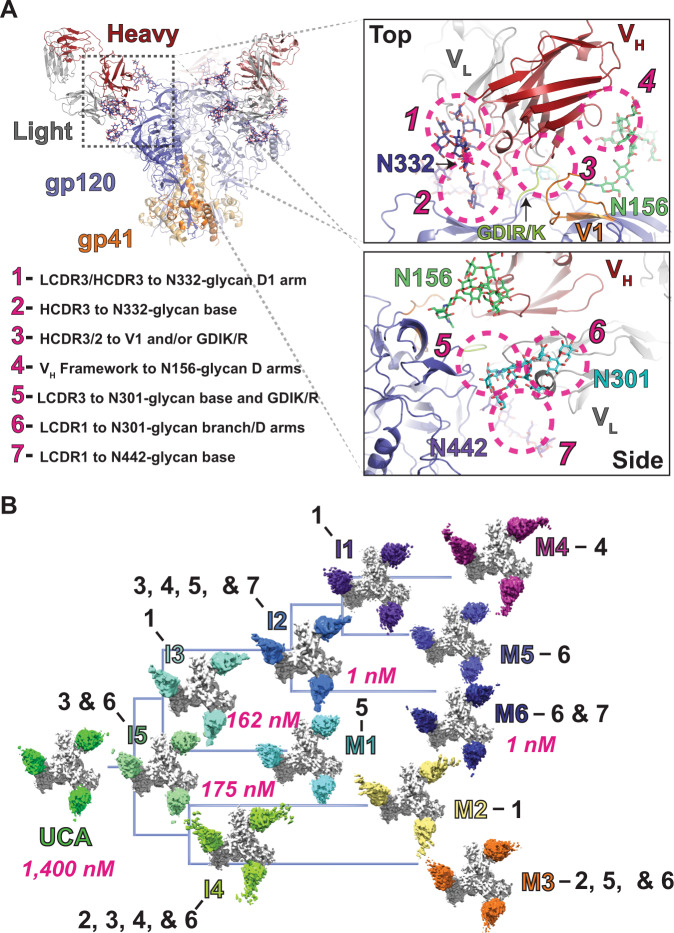
T*he V3-glycan targeting DH270 broadly neutralizing antibody clone*. **A** (upper left) A DH270 antibody Fab bound HIV-1 Envelope (Env) ectodomain highlighting the gp120 (blue) and gp41 (orange) subunits. Antibody Fab heavy and light chains are colored red and grey, respectively, with epitope glycans depicted in stick representation. (right) Zoomed-in, top and side views of the paratope-epitope contacts. Dashed circles indicate distinct contact regions. (lower left) Number indicators identify contact sites highlighted by the dashed circles in the panels to the right. **B** The inferred DH270 clone phylogenetic tree depicting cryo-EM maps determined for each clonal member Fab bound to the CH848 d949 trimer (white). The clonal member names are colored according to the Fab color of each map. Black numbering indicates locations of consequential mutations in each antibody according to sites listed in **A**. Affinities for antibodies on the path leading to the most broad and potent mature form, M6, are indicated in pink. Intermediate clonal members are denoted with an I while mature antibodies are denoted with an M.

The structures of several DH270 antigen-binding fragments (Fabs) have been previously determined, including inferred DH270 UCAs, as well as the mature DH270.3, DH270.5, and DH270.6 antibodies that were isolated from the CH848 individual. Additionally, structures have been determined of a DH270.1 single-chain variable fragment (scFv), a Mannose associated DH270.3, a V3-glycan peptide associated DH270.6 scFv, and soluble Env ectodomain bound DH270 UCA and DH270.6 Fabs^20,21,30^. While the V3-glycan associated DH270.6 structure clarified the role of individual residues critical for neutralization breadth, it did not reveal the structural pathway underlying the evolution of this breadth, nor show how breadth and potency were affected by mutations in different DH270 clades and subclades. We therefore sought to determine the impact of mutations at each stage of maturation by determining cryo-EM structures of HIV-1 Env associated DH270 clonal member Fabs from each antibody ancestral intermediate, and all mature antibody forms (Fig. 1 and Supplemental Figs. 1 and 2). A soluble SOSIP trimer derived from a virus isolated from the CH848 individual at day 949 (referred to here as the d949 Env) after transmission was used for preparing complexes with DH270 clone Fabs for structural studies. This Envelope was selected for its temporal proximity to DH270 lineage appearance in the infected patient and because it interacts with all DH270 lineage members. With the Env constant, all observed structural differences can be attributed to antibody amino acid differences. Map resolutions ranged from 5.3 to 3.3 Å with local resolutions at the Fab-Env interface in the range of 5–3 Å (Supplemental Figs. 2-4, Tables 1 and 2).

**Table 1:** cryo-EM data collection, map, and model refinement statistics

	(DH270.UCA)	(DH270.UCA.G57R-CH848.10.17DT)	(DH270.I5.6-CH848.10.17)	(DH270.I4.6-CH848.10.17)	(DH270.I3-CH848.10.17)	(DH270.I2-CH848.10.17)	(DH270.I1.6-CH848.10.17)	(DH270.1-CH848.10.17)	(DH270.2-CH848.10.17)	(DH270.3-CH848.10.17)
**Data Collection**										
Microscope	FEI Titan Krios									
Detector	Gatan K3									
Magnification	105000									
Voltage (kV)	300									
Electron dose (e^-^/Å^2^)	71	69	53	60	60	60	60	60	60	60
Pixel Size (Å)	1.06	1.066	1.066	1.08	1.066	1.066	1.04	1.066	1.066	1.066
Defocus Range (µm)	~0.8-4.3	~0.7-4.1	~0.5-4.1	~0.5-3.1	~0.5-3.5	~0.5-3.5	~0.5-4	~1.5-3.5	~0.5-4	~0.5-3.5
**Reconstruction software cryoSPARC**										
Symmetry	C3	C3	C3	C3	C3	C3	C3	C3	C3	C3
Particles	120981	208426	101922	338,934	67110	39612	65522	125676	24465	76274
Box size (pix)	384	384	384	300	384	384	384	384	384	384
Resolution (Å) (FSC0.143)^a^	4.0	3.3	3.9	4.2	4.3	3.5	3.9	3.6	4.1	3.4
**Coordinate Refinement (Phenix)**^b^										
Protein residues	2496	2490	2490	2490	2490	2490	2490	2490	2490	2490
**R.M.S. deviations**										
Bond lengths (Å)	0.006	0.006	0.006	0.007	0.006	0.006	0.006	0.013	0.006	0.006
Bond angles (°)	0.908	0.892	0.909	0.987	0.898	0.990	0.885	1.159	0.926	0.933
**Validation**										
Molprobity score	1.42	1.48	1.39	1.48	1.57	1.56	1.42	1.44	1.54	1.52
Clash score	2.15	2.86	2.30	2.56	3.06	3.95	2.59	2.91	2.54	2.98
Favored rotamers (%)	99.77	99.53	99.72	99.63	99.67	99.21	99.86	99.72	99.53	99.68
EMRinger Score	2.12	2.06	2.28	1.81	2.73	2.53	2.61	2.23	1.39	1.63
**Ramachandran plot**										
Favored regions (%)	93.41	94.04	94.4	93.11	92.34	94.48	94.44	94.77	91.65	93.31
Disallowed regions (%)	0.2	0.36	0.12	0.24	0.24	0.16	0.36	0.32	0.49	0.28

^a^ Resolutions are reported according to the FSC 0.143 gold-standard criterion.

^b^ Statistics are reported for the protein residues within the complex excluding the antibody constant domains.

**Table 2:** cryo-EM data collection, map, and model refinement statistics

	(DH270.4-CH848.10.17)	(DH270.5-CH848.10.17)	(DH270.6-CH848.10.17)	(VRC01-CH848.0526.25)	(VRC01-CH848.10.17)	(DH270.6-CH848.0526.25)	(VRC01-CH848.0836.10)	(VRC01-CH848.0358.80)
**Data Collection**								
Microscope	FEI Titan Krios							
Detector	Gatan K3							
Magnification	105000							
Voltage (kV)	300							
Electron dose (e^-^/Å^2^)	60	60	53.15	69.98	60	60	60	60
Pixel Size (Å)	1.066	1.066	1.066	1.066	1.066	1.066	1.066	1.066
Defocus Range (µm)	~0.8–4.3	~0.7–4.1	~0.5–4.1	~0.5–3.1	~0.5–3.5	~0.5–3.5	~0.5–3.5	~0.5–3.5
**Reconstruction Software cryoSPARC**								
Symmetry	C3	C3	C3	C3	C3	C3	C3	C3
Particles	107890	76211	106823	252873	35839	119542	28746	46336
Box size (pix)	384	384	384	384	384	384	384	384
Resolution (Å) (FSC0.143)^a^	3.3	4.0	3.82	4.0	4.5	3.9	4.6	4.9
**Coordinate Refinement (Phenix)**^**b**^								
Protein residues	2490	2496	2490	2514	2457	2574	2505	2523
**R.m.s. deviations**								
Bond lengths (Å)	0.006	0.008	0.006	0.004	0.005	0.006	0.004	0.006
Bond angles (°)	0.915	1.100	1.055	0.845	0.894	1.041	0.949	0.997
**Validation**								
Molprobity score	1.57	1.71	1.71	1.53	1.80	1.76	1.71	1.90
Clash score	3.13	4.02	4.37	2.96	5.01	4.26	4.65	6.60
Favored rotamers (%)	99.72	99.68	99.30	99.77	99.81	99.73	99.59	100
EMRinger Score	1.95	0.36	1.74	1.66	1.65	1.49	1.31	0.56
**Ramachandran plot**								
Favored regions (%)	92.46	90.94	91.93	93.14	90.56	89.78	92.51	90.15
Disallowed regions (%)	0.32	0.44	0.41	0.24	0.41	0.35	0.12	0.24

^a^ Resolutions are reported according to the FSC 0.143 gold-standard criterion.

^b^ Statistics are reported for the protein residues within the complex excluding the antibody constant domains.

Mapping the mutations of the DH270 clone on the cryo-EM structures revealed clustered sites that are sequentially mutated along the B cell clonal tree (Fig. 1A). These clusters mapped to seven distinct sites of antibody/Env contact: (1) interaction of the distal D1 arm of the Env N332-glycan with the cleft formed between the Fab variable heavy and light chain segments (V_H_/V_L_) involving the light-chain complementarity determining region two (LCDR2) and heavy-chain complementarity determining region three (HCDR3), (2) interaction of the N332-glycan GlcNac base with the antibody HCDR3 region, (3) contact of the Env V1 loop, and of the conserved GDIR/K motif in the V3 loop with the Fab HCDR3 and HCDR2 regions, (4) interaction of the D arms of the Env N156-glycan with the antibody framework region, (5) interaction of the Env N301-glycan base GlcNac-1 and residues around the V3 GDIR/K motif with the Fab LCDR3 region, (6) contact of the Env N301-glycan branch point and D arms with the Fab LCDR1/V_L_ N-terminus site, and (7) contact of the Env N442-glycan with Fab LCDR1 region (Fig. 1A). Together, these comprise the entirety of the interactive surface available to the DH270 clone antibodies.

The cryo-EM reconstructions were resolved at the Ab/Env interfaces to allow atomic model building. We did not observe any clear patterns of improvement in the interface resolutions with enhanced interaction strength. This could be due to variability in the cryo-EM datasets, as well as reductions in thermal stability in DH270 lineage during affinity maturation^6^.

Clear patterns of mutations emerged along the DH270 clonal tree at the Env interactive sites described above that were suggestive of the DH270 clone evolving to resolve specific, sequential structural bottlenecks on the path of affinity maturation (Fig. 1B). The first intermediate, I5, acquired mutations that strengthened interactions at clusters 3 and 6 by improving protein-protein contacts as well as the interactions of the antibody with the N301 glycan, resulting in ~8-fold improved binding affinity to the d949 Env relative to the UCA. After intermediate I5, the clone splits into two distinct paths along a clade defined by the I3 intermediate and a clade defined by the I4 intermediate. This consequential split leads to the acquisition of greater neutralization breadth and potency in the I3 clade, leading to the DH270.6 bnAb (Fig. 1B)^20^. Both clades acquired mutations around the N332-glycan interaction site but at differing positions. The I3 intermediate improves interaction with the distal N332-glycan D1 arm (cluster 1) while the I4 intermediate improves interaction with the N332-glycan GlcNac base (cluster 2). Each intermediate then splits into two subclades, revealing a conserved Env target for contact optimization by three of the four mature/intermediate antibodies. These include the I4 clade mature antibody DH270.3, I3 clade mature antibody DH270.1, and intermediate antibody I2, which all show several mutations clustered in or adjacent to the antibody LCDR3 region, presumably improving interactions with the Env N301-glycan base (cluster 5). The I4 clade mature antibody DH270.2 acquired mutations focused on cluster 1, improving interactions at the N332-glycan D1 arm in a manner similar to the I3 intermediate. The I2 intermediate acquired additional mutations at clusters 3, 4, and 7, with mature DH270.6, the most broad and potent antibody of the clone, further modifying interactions at cluster 7. A minimally mutated DH270 antibody (minDH270) was designed^29^ where only 12 of the 42 mutations in DH270.6 were needed to reach ~90% of the breadth and potency of DH270.6. Importantly, the mutations included only those that appeared in I5, I3, and I2. Consistent with this observation, mutations after I2 occurred at more distal sites and their effects were less clear compared to the mutations that appeared in the previous members of the DH270 clone, as described below. The DH270.5 and DH270.6 antibodies acquired several mutations near the LCDR1/V_L_ N-terminus where interactions with the N301-glycan D arms may occur. The cryo-EM reconstructions in this region were poorly resolved, precluding a precise definition of these interactions. Together, these results indicate that maturation of the DH270 clone involved sequential affinity gain at distinct sites, and that specific structural solutions were necessary for affinity gain and development of neutralization breadth. This is exemplified in the dichotomy where acquisition of mutations in the antibody LCDR3 region led to neutralization breadth gain in DH270.1 and in the I2 intermediate, but loss in neutralization breadth in DH270.3.

### UCA to I5: Early mutations in clone development set the stage for downstream maturation

**Fig. 2:**
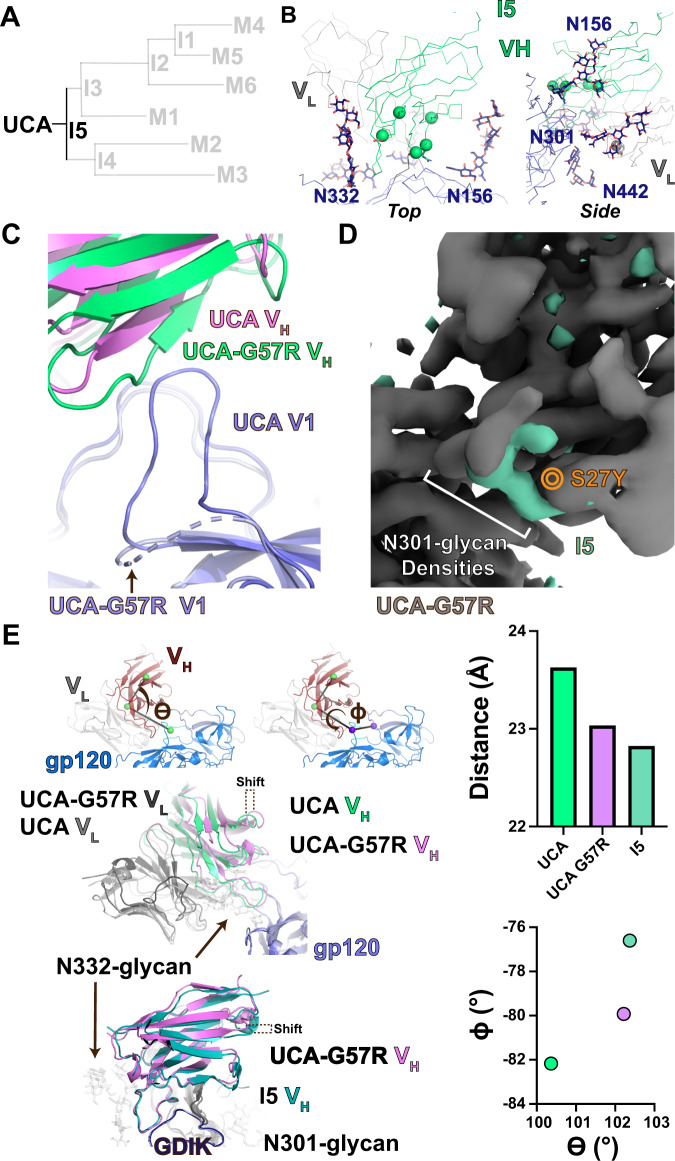
*The UCA to I5 transition*. **A** DH270 clonal tree highlighting the UCA and I5 intermediate antibodies. **B** Top and side views of the I5 contact site. Alpha-carbons (Cα) of heavy and light chain mutation are presented as spheres. **C** Top view of the UCA and UCA+G57R heavy chain contacts with gp120 highlighting the shifts in the antibody position and in the Env gp120 V1 loop. The dashed cartoon representation for the UCA-G57R bound Env V1 connects residues 133 and 153 between which map density for V1 is weak. **D** Gaussian filtered map of the UCA+G57R Fab bound trimer complex (grey) overlaid with a filtered and subtracted map of the I5 Fab bound trimer complex (green). The location of the S27Y mutation on the maps is indicated in orange. **E** (upper left) Contact angle theta and dihedral phi with positions of the centroids used for calculations indicated with spheres. (middle left) Alignment (gp120 only) of UCA and UCA+G57R bound state structures highlighting the shift in antibody disposition about the theta angle. (lower left) Alignment (gp120 only) of UCA+G57R and I5 bound state structures highlighting the shift in antibody disposition about the phi dihedral. (upper right) Antibody Fab V_H_/V_L_ centroid distance from the gp120 epitope centroid. (lower right) Theta vs. phi plot for the UCA, UCA+G57R, and I5 structures. The colors of the data points match the respective heavy chain colors in the structures.

We next studied the specific structural arrangements underlying maturation at each stage, first examining mutations in the I5 intermediate (Fig. 2A, B). We previously determined structures for the DH270 UCA and the mature DH270.6 Fabs bound to the CH848 d949 Env trimer with V1 loop glycans at positions 133 and 138 removed^21^. In the UCA-bound structure the V1 loop interacts with the antibody, the V3 GDIK motif and adjacent V3 residues, while in the DH270.6-bound Env the V1 loop was displaced from this position such that it no longer interacts with the V3 GDIK motif. This displacement was attributed to the V_H_ G57R mutation that occurs in the first intermediate^21^. To visualize the V1 loop conformation when not bound to an antibody at the V3 glycan bNAb epitope, we determined the structure of the d949 SOSIP Env in complex with the CD4 binding site antibody VRC01. We found that the conformation of the unbound V1 loop resembled that of the UCA-bound V1 loop (Supplemental Fig. 6A). We further determined the structure of DH270.UCA incorporating the V_H_ G57R mutation in complex with the CH848 d949 SOSIP Env (Fig. 2C and Supplemental Fig. 6B). The V1 loop in the UCA+G57R structure was displaced from its position observed in its free and UCA-bound forms where it interacted with and shielded the V3 GDIK motif (Supplemental Fig. 6C). The displacement of the V1 loop in the UCA+G57R structure is accompanied by a marked shift in the antibody orientation, rotating the Fab V_H_/V_L_ toward the space previously occupied by the V1 loop, drawing the antibody closer to the Env (Fig. 2C and Supplemental Fig. 6D). Comparing the structure of the UCA+G57R-bound complex with the I5-bound complex revealed further shifts in the orientation of the antibody orchestrated by the other acquired mutations in the I5 intermediate. The V_L_ S27Y substitution near the β-Mannose branchpoint of the N301-glycan is the likely culprit, altering the orientation of N301 glycan in the I5-bound complex relative to the UCA+G57R bound complex (Fig. 2D).

We next quantified these shifts in antibody orientation using vector-based angles between the antibody F_v_ and its gp120 epitope. Centroids for the epitope, the F_v_, and an anchor point in the V_H_ were used to determine the distance between the F_v_ and its epitope and angle (theta), with an additional anchor point in the epitope added to examine rotation (phi) of the antibody relative to the epitope (Fig. 2E). Theta and phi describe the antibody angle of approach with reference to the epitope, allowing close inspecting of differences in antibody disposition as mutations accumulate in the clone. The distance between the antibody and the epitope was reduced by ~0.5 Å in the UCA+G57R bound structure relative to the UCA-bound structure, with further minor reductions observed in I5 associated with the additional I5 mutations (Fig. 2E). Comparison of the angle and dihedral dispositions of the UCA and UCA+G57R structures showed a rotation in each of ~2°. The I5 intermediate showed further rotation about the phi dihedral in the direction of the S27Y mutation without additional changes in the theta rotation angle. Together, these results demonstrate that early mutations in the DH270 clone facilitate improved contacts, shift the position of the antibody relative to the bound Env, and alter Env conformation, particularly of the V1 loop and N301 glycan, with the angular and conformational changes further strengthening antibody-Env interactions.

### The I5 path split: Mutations in I3 set the path toward broad neutralization

**Fig. 3:**
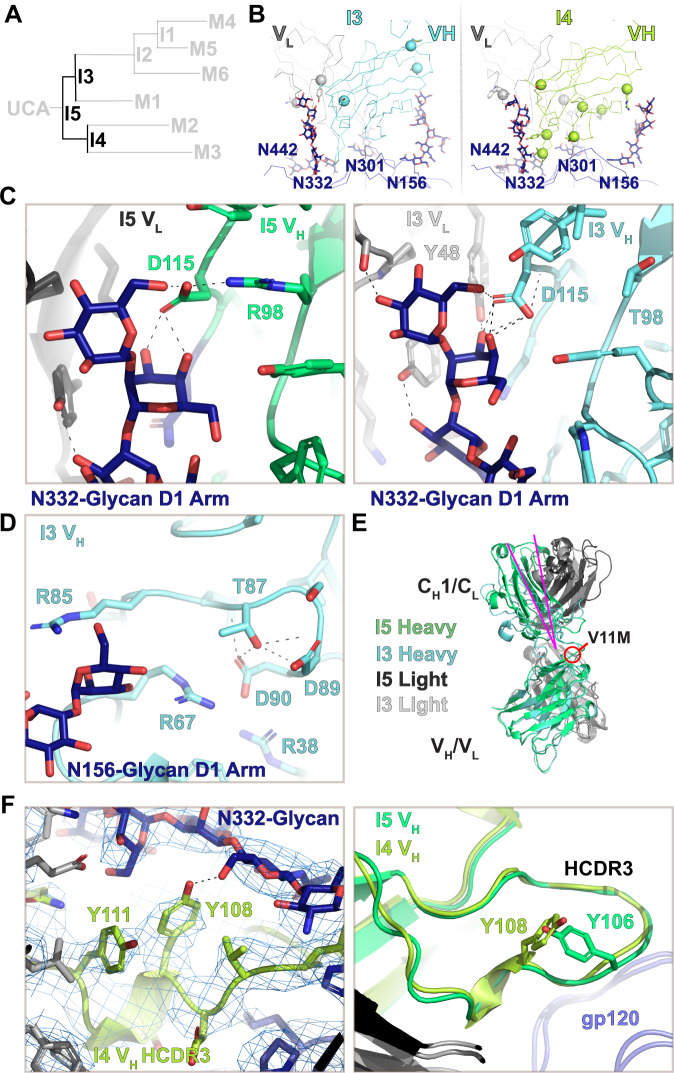
*The I5 to I3 and I4 path split*. **A** DH270 clonal tree highlighting the I5, I3, and I4 intermediate antibodies. **B** Top views of the I3 and I4 contact sites. Alpha-carbons (Cα) of heavy and light chain mutations are presented as spheres. **C** (left) The N332-glycan D1 arm contact with the I5 V_H_/V_L_ cleft. (right) The N332-glycan D1 arm contact with the I3 V_H_/V_L_ cleft. **D** Structure of the I3 intermediate at the R87T site near potential N156-glycan contacts. **E** Aligned I5 and I3 V_H_ domains highlighting the change in elbow angle (magenta). **F** (left) Map and fit coordinates of the new N332-glycan contact formed at the I4 HCDR3 S108Y mutation site. (right) Aligned I5 and I4 V_H_ domains showing HCDR3 loop arrangements.

We next examined the early development of two distinct clades in the DH270 clone from the I5 intermediate (Fig. 3A, B). The I3 intermediate that occurs after I5 in the DH270.6 clone incorporates heavy chain mutations V11M, R87T, and the improbable R98T mutation, and light chain mutations L48Y and S54N. The cryo-EM reconstruction of the I3 Fab bound Env was determined to a resolution of 4.5 Å. The V1 loop conformational change induced by the V_H_ residue R57 was retained as was the interaction of V_L_ residue Y27 with the N301-glycan (Fig. 3B and Supplemental Fig. 7A, B). No major differences were observed in the I5 and I3 Fab bound Env structure gp120 and gp41 subunits (RMSDs ~0.9 Å; Supplemental Fig. 7C). As previously described^30^, the V_L_ L48Y and V_H_ R98T mutations introduce new hydrogen bonding contacts, with the Y48 hydroxyl interacting with the N332-glycan, and the release of D115 through the R98T mutation increasing the number of D115 hydrogen bond interactions with the N332-glycan (Fig. 3C and Supplemental Fig. 7D). Additionally, the I3 V_H_ R98T substitution introduced a hydrogen bond between the side chains of residues T98 and the V_H_ Y27 (Supplemental Fig. 7E), thus bolstering internal stability of the antibody structure by compensating for the loss of the cation-π interaction of R98 with Y27. The V_H_ R87T and V_L_ S54N mutations are distant from the gp120-interactive region of the antibody. Close examination of the local regions of these mutations suggested potential roles of these substitutions in augmenting internal stability of the antibody. The V_H_ R87T substitution relieves electrostatic strain contributed by three spatially clustered arginines, R67, R85 and R87 (Fig. 3D and Supplemental Fig. 7F). Notably, a second arginine in this cluster, R85, is mutated to serine at the next step as I3 transitions to I2. Moreover, the T87 side chain makes a hydrogen bond with main chain nitrogen of residue V_H_ D89, thus augmenting stability of local structure. The effect of V_L_ S54N was more subtle with N54 appearing to stabilize interaction with an adjacent beta strand via hydrogen bonding of its side chain with the main chain of light chain residue 66 (Supplemental Fig. 7G).

The I5 and I4 antibody structures were similar, and as predicted by previous molecular dynamics simulations^6^, the V11M mutation in I3 induced a marked shift in the antibody Fab elbow hinge (Fig. 3E). Unlike I3, the I4 intermediate did not acquire an elbow mutation and consequently did not show a distinct shift in the elbow region (Supplemental Fig. 7H). As in I3, the R57 residue in I4 showed evidence of shifting the position of the V1 loop (Supplemental Fig. 7I). However, additional density was observed in this region that is consistent with a GDIK coupled V1 loop, suggestive of multistate behavior. The I4 intermediate acquired a larger number of V_H_ mutations including two in the HCDR3, Y106V, and S108Y that essentially shift the position of the tyrosine from residue 106 to 108. The hydroxyl group in I4 Y108 formed a hydrogen bond with the N332 GlcNac-2 (Fig. 3F left). The HCDR3 backbone conformation was not altered by these mutations (Fig. 3F right). The I4 S84R mutation is in the vicinity of the N156-glycan distal sugar units. Densities for the side chain and this portion of the N156-glycan were, however, not visible in the cryo-EM reconstruction (Supplemental Fig. 7J, K). The remaining mutations in the I4 heavy chain at positions G49A, N54T, Q62R, and Y116S play no clearly decipherable role in the interaction with Env or in modifying the antibody structure. The light chain contains two non-paratope mutations, R56W and V100I. No changes were observed around V100I position relative to I5 or I3. Residue R59W of the I4 light chain that is adjacent to the LCDR2 region displayed a modest rearrangement that shifts the position of distal N332-glycan interactive residues (Supplemental Fig. 7L). This was not associated with apparent changes in antibody interactions with the glycan though it may confer stabilization of the LCDR2 loop and therefore the glycan interaction. These observations show that at this stage of development, interactions with the N332-glycan were the focus of optimization in both clades of the DH270 clonal tree.

### Maturation after I4: DH270.2 and DH270.3 attempt improvements in N332-glycan interactions or GDIK and N301-glycan interactions

**Fig. 4:**
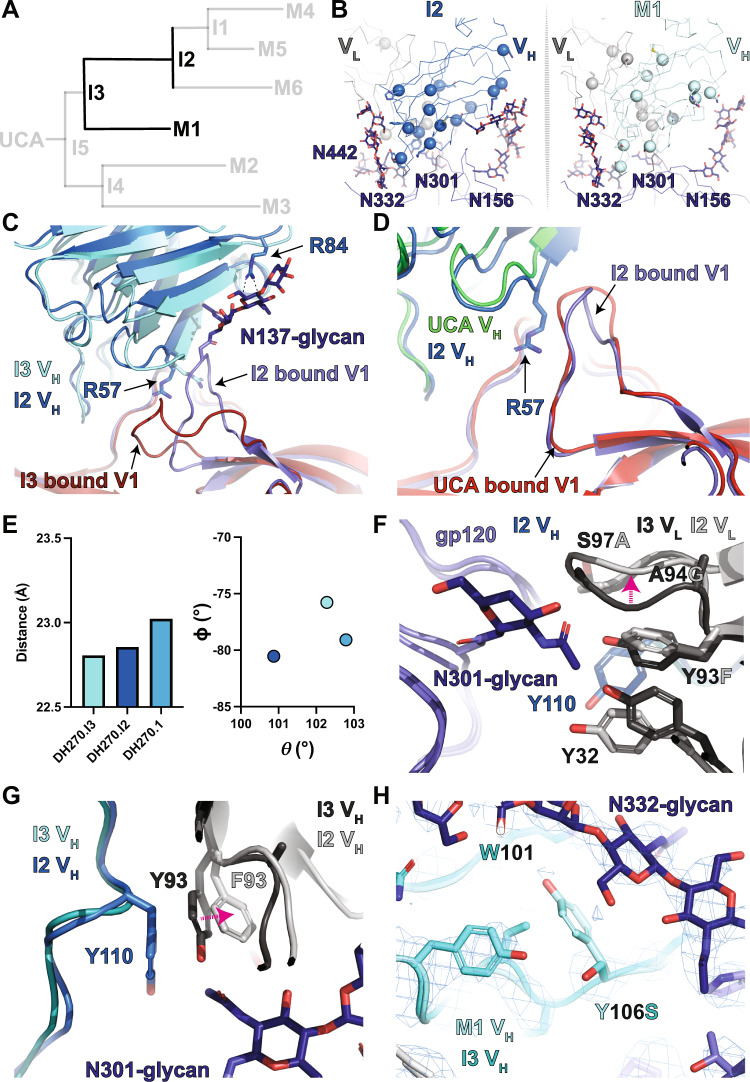
*The I3 to I2 and DH270.1 path split*. **A** DH270 clonal tree highlighting the I3 and I2 intermediate antibodies and the mature DH270.1 (M1) antibody. **B** Top views of the I2 and DH270.1 contact sites. Alpha-carbons (Cα) of heavy and light chain mutations are presented as spheres. **C** Alignment (gp120 only) of I3 and I2 bound state structures highlighting the shift in gp120 V1 loop arrangement and contact between the I2 R84 side chain and the N137-glycan. **D** Alignment (gp120 only) of UCA and I2 bound structures highlighting conformational similarity between the gp120 V1 loops. **E** (right) Antibody Fab V_H_/V_L_ centroid distance from the gp120 epitope centroid. (left) Theta vs. phi plot for the I3, I2, and DH270.1 structures. The colors of the data points match the respective heavy chain colors in the structures. **F** Side view comparison of the LCDR3 region epitope contact between I3 and I2 highlighting the shift in LCDR3 conformation (magenta arrow). **G** Top view comparison of the LCDR3 region epitope contact between I3 and I2 highlighting the shift in the aromatic residue 93 rotamer (magenta arrow). **H** Alignment (gp120 only) of I3 and DH270.1 bound state structures with DH270.1 map highlighting the Y106S mutation.

The I4 clade leads to mature antibodies DH270.2 and DH270.3 (Fig. 4A, B). DH270.2 showed greater breadth and potency than DH270.3 with each neutralizing ten and fifteen members from a 24-virus heterologous panel, respectively, although both displayed weaker neutralization breadth and potency compared to the I3 clade mature antibodies that neutralize sixteen to seventeen members of the heterologous panel^21^. Alignment of the Env-bound structures revealed distinct shifts in the orientation of each antibody compared to the I4 intermediate (Fig. 4C and Supplemental Fig. 8A). Inspection of the theta and phi angles indicated DH270.3 had predominantly shifted its rotation about the epitope (Fig. 4D). The most notable mutation in DH270.3 was the light chain Y27S reversion. A clear shift in the position of the N301-glycan was observed relative to the I4 intermediate (Fig. 4E and Supplemental Fig. 8B). Consistent with the observed rotation of the bound antibody in the I5/Env complex relative to the UCA+G57R/Env complex, the Y27S reversion resulted in DH270.3 occupying an orientation matching UCA+G57R (Fig. 4F). This reversion of antibody orientation suggests that the S27Y substitution, first acquired in I5, is the driver of the antibody rotation and N301-glycan re-orientation that was observed in going from the UCA to I5.

A cluster of mutations, including an improbable V_H_ G110Y mutation, occurs in and around the DH270.3 LCDR3, near the contact between a loop adjacent to the V3 GDIK motif and the N301-glycan GlcNac base. Additional mutations, F93S, A94T, G95N, and S97A, result in remodeling of the N301-glycan interaction, shifting the position of the LCDR1 Y32 side chain for interaction with the first N301-GlcNac and interaction of N95 with the second N301-GlcNac (Fig. 4G). In addition to the angular shift and LCDR3-related mutations, a G103S mutation in HCDR3 adds a hydrogen bond with the N332-glycan GlcNac-2 N-acetyl moiety (Supplemental Fig. 8C). While DH270.3 mutations optimized interactions with the N301-glycan, the DH270.2 mutations focused instead on the D1 arm N332-glycan interaction. Of the three heavy chain mutations R98K, W101Y, and D115N, the D115N mutation showed the clearest shift in interaction. Loss of the negative charge at D115N allowed the asparagine side chain to shift away from the positively charged K98 by ~1.6 Å (D and N Cγ to R Nε or K Cε) to form an extensive hydrogen bonding network with the N332-glycan D1 arm (Fig. 4H). This mirrored the R98T mutation in I3, where D115 was freed from its interaction with the positively charged V_H_ residue R98 to engage the N332-glycan. Together, analysis of the Env-bound DH270.2 and DH270.3 structures suggest each followed different paths of development with each targeting distinct sites. The greater breadth of the DH270.2 antibody compared to DH270.3 is likely a combined effect of improvement in the N332-glycan interaction in DH270.2 and the Y27S reversion in DH270.3.

### Maturation after I3: A complex series of mutations in I2 resolve epitope clashes

**Fig. 5:**
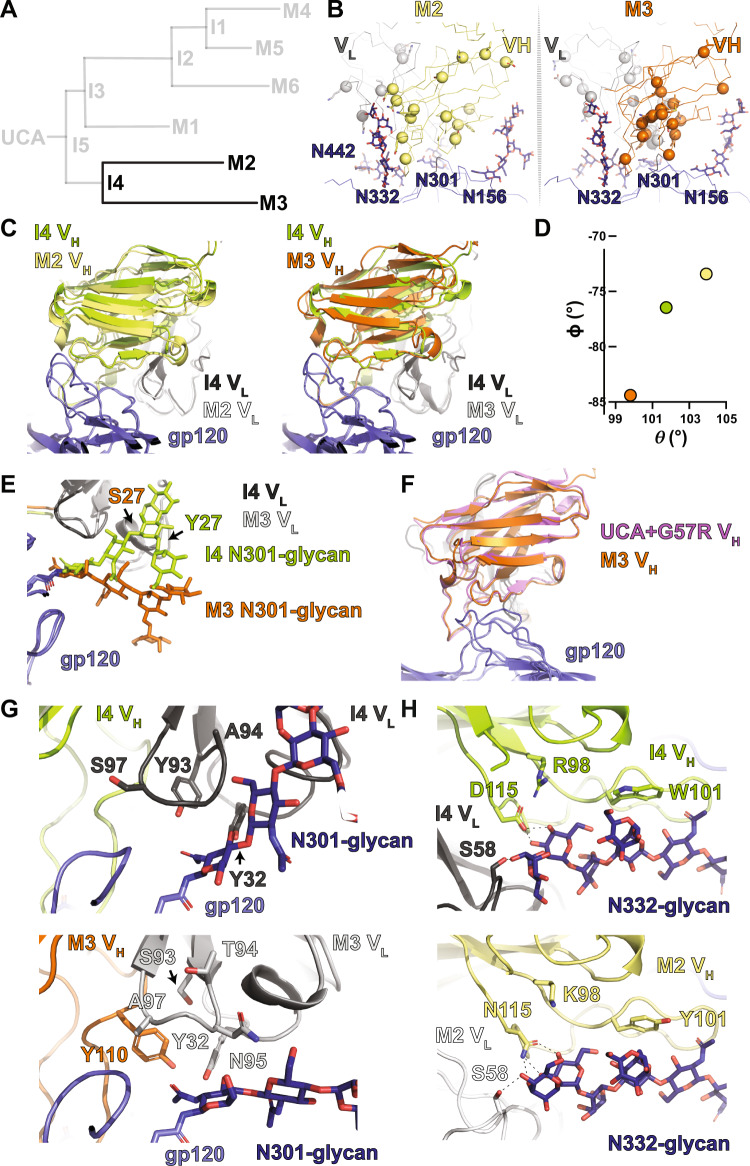
*The I4 to mature DH270.2 and DH270.3 transition*. **A** DH270 clonal tree highlighting the I4 intermediate antibody and the mature DH270.2 (M2) and DH270.3 (M3) antibodies. **B** Top views of the DH270.2 and DH270.3 contact sites. Alpha-carbons (Cα) of heavy and light chain mutations are presented as spheres. **C** (left) Alignment (gp120 only) of I4 and DH270.2 bound state structures highlighting the shift in antibody disposition (right) Alignment (gp120 only) of I4 and DH270.3 bound state structures highlighting the shift in antibody disposition. **D** Theta vs. phi plot for the I4, DH270.2, and DH270.3 structures. The colors of the data points match the respective heavy chain colors in the structures. **E** Structure overlay of the I4 and DH270.3 N301-glycans showing their change in disposition and the location of the S27 or Y27 residues on the antibody light chain. **F** Alignment (gp120 only) of UCA+G57R and DH270.3 bound state structures indicating similar bound state arrangements. **G** Comparison of the LCDR3 contact site with the gp120 protein and N301-glycan between the I4 (upper) and DH270.3 (lower) structures. **H** Comparison of the N332-glycan D1 arm antibody contacts between the I4 (upper) and DH270.2 (lower) structures.

The next stage of development in the I3 clade of the DH270 clone led to the most substantial gains in Env binding affinity (Fig. 1B) and HIV-1 neutralization breadth^20^. The I3 intermediate splits into the I2 intermediate and the mature DH270.1 antibody, both of which acquire mutations that are primarily focused on the LCDR3 region (Fig. 5A, B). A notable feature in the cryo-EM reconstruction of the I2-Env complex is the well-resolved V1 loop with an altered configuration. Well-resolved density was also observed for the V1 loop N137-glycan, which contacted the I2 heavy chain S84R mutation site (Fig. 5C and Supplemental Fig. 9A). This mutation also occurred in the I4 intermediate which showed similar, albeit less clear, densities for a V1 loop that was coupled with the V3 GDIK motif. Re-orientation of the V1 loop shifted R57 toward the V3 GDIK motif, resulting in its interaction with D325 (Supplemental Fig. 9B). This V1 loop orientation was similar to the UCA-bound trimer structure with a kink in the loop introduced to accommodate R57 (Fig. 5D). Quantification of the antibody orientation showed a rotation of the I2 V_H_/V_L_ away from the V1 loop to accommodate this loop rearrangement (Fig. 5E).

The I2 intermediate and DH270.1 both acquire several LCDR3 modifying mutations like those observed in DH270.3 (Fig. 5F, G and Supplemental Fig. 9C, D). In DH270.1 several mutations occurred at the LCDR3 base that were distal to the epitope, and together, they likely stabilized the LCDR3 region (Supplemental Fig. 9E, F). Similar mutations occur in the I2 intermediate, most notably an F101C mutation spatially adjacent to the LCDR3 base that results in the formation of an additional disulfide bond in the antibody between residues 91 and 101 (Supplemental Fig. 9D). This mutation is among the previously described minimal set of mutations needed to reach ~90% of DH270.6 breadth^29^. A considerable number of mutations in and affecting LCDR3 were also a part of the minDH270 construct and have distinct effects on epitope interaction in the I2 intermediate bound structure. These included the V_L_ Y93F, A94G, and S97A that work in concert with V_H_ G110Y to cause a rearrangement in the LCDR3 region, creating a pocket in which the N301 GlcNac-1 N-acetyl moiety rests (Fig. 5F). An important impact of the improbable HCDR3 G110Y mutation in creating this pocket is the shifting of the F93 rotamer via steric occlusion (Fig. 5G). Additionally, an HCDR3 Y106S mutation occurs in DH270.1 that resulted in two effects. First, the loss of a hydrogen bond between the side chain of Y106 with the main chain of W101 and, second, the release of the interaction of the Y106 side chain with the N332 glycan; together these may act to modulate the interaction of glycan N332 with HCDR3 by allowing it to engage the W101 side chain more effectively (Fig. 5H). These results, together with the observed LCDR3 changes in DH270.3, suggest modifications of the interaction at this site were essential for affinity maturation.

### Maturation after I2: Maturation shifts to the epitope periphery

**Fig. 6:**
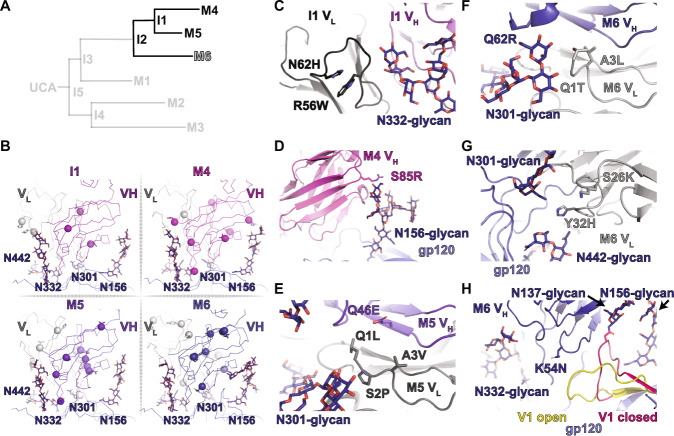
*The I2 to I1, DH270.4, DH270.5, and DH270.6 transition*. **A** DH270 clonal tree highlighting the I2 and I1 intermediate antibodies and the mature DH270.4 (M4), DH270.5 (M5), and DH270.6 (M6) antibodies. **B** Top views of the I1, DH270.4, DH270.5, and DH270.6 contact sites. Alpha-carbons (Cα) of heavy and light chain mutations are presented as spheres. **C** Structure of the I1 V_H_/V_L_ contacts with the N332-glycan D1 arm showing the positions of the V_L_ N62H and R56W mutations. **D** Structure of the DH270.4 heavy chain showing the position of possible contact between the S85F mutation and the N156-glycan. **E** Structure of the DH270.5 V_H_/V_L_ mutations proximal to the N301-glycan. **F** Structure of the DH270.6 V_H_/V_L_ mutations proximal to the N301-glycan. **G** Structure of the DH270.6 V_L_ mutations proximal to the N442-glycan. **H** Structure of the DH270.6 V_H_ shows two different states of the gp120 V1 loop.

From the I2 intermediate the clone splits into two paths, both culminating in similar neutralization breadth, albeit with DH270.6 having the greatest potency (Fig. 6A, B)^20^. The upper path from I2 leads to the I1 intermediate and then onto the mature DH270.4 and DH270.5 antibodies. Mutations in this I1 subclade were generally further from the antibody-antigen interactive surface with less clear structural impacts. In I1, two mutations with possible impacts on the LCDR2 loop, N62H, and R65W, could affect N332-glycan D1 arm interactions (Fig. 6C and Supplemental Fig. 10A). This loop was, however, poorly resolved, limiting our ability to determine what, if any, impact these mutations have. In DH270.4, a V_H_ S85R mutation is positioned for interaction with the N156-glycan (Fig. 6D and Supplemental Fig. 10B). However, as was the case for the I1 intermediate mutations, this region is poorly resolved and the interaction cannot, therefore, be confirmed. Although the N301-glycan D arm was not well-defined in density, the V_H_ and V_L_ of both DH270.5 and DH270.6 acquired several mutations in proximity to the N301-glycan D arm (Fig. 6E, F and Supplemental Fig. 10C, D). Densities consistent with both the V3 GDIK coupled and uncoupled V1 loop states were apparent in the DH270.6 bound trimer (Fig. 6H and Supplemental Fig. 10E, F). Additional mutations in DH270.6 occurred near the N442-glycan and the V1 loop configuration that was displaced from the V3 GDIK motif (Fig. 6G, H). Examination of these structures indicates mutations after I2 occurred primarily at regions more distal to the epitope, consistent with the ability of I2 to neutralize most of the viruses neutralized by DH270.6.

Together, structural determination of the entire DH270 clone revealed an intricate process through which HIV-1 neutralization breadth was developed in the V3-glycan supersite directed DH270 clone, with early improbable G57R and S27Y mutations of the I5 intermediate altering antibody orientation and conferring heterologous neutralization breadth. Development of two distinct clades from I5 to the I3 and I4 intermediates resulted in both acquiring improvements in N332-glycan interaction further highlighting the importance of N332-glycan engagement for the DH270 clone. Each, however, targeted distinct sites of the glycan, with I3 stabilizing the distal D1 arm and I4 stabilizing the GlcNac base. Downstream maturation from I4 to DH270.2 suggests this was not an ideal solution to acquiring N332-glycan interaction, as DH270.2 acquires mutations like those in I3 for the N332-glycan D1 arm. Post-I3, leading to I2 and DH270.1 along with DH270.3, all show a considerable number of mutations in the LDCR3 and adjacent residues. While DH270.3 acquires several mutations critical for neutralization, including G110Y and S94A, the selection of the Y27S reversion may have limited its breadth. Where DH270.1 acquired stabilizing LCDR3 mutations, I2, which developed considerable neutralization breadth, modified the LCDR3 conformation, stabilizing a N301-glycan base contact point. This was mediated by at least five residues, each of which alone may have provided limited affinity gain. In the case of DH270.3, had it first stabilized the N332-glycan D1 arm, the limited gains associated with optimizing LCDR3 site might have been tractable. Paratope modifications downstream from the I2 intermediate were distant from the primary epitope and likely acted to fine tune affinity, breadth, and potency. The minDH270 antibody^29^ required only mutations from I5 to I2, consistent with this observation. These results show that selection during affinity maturation can be narrowed to a series of structural bottlenecks with the developing antibody clone demonstrating optimal and suboptimal solutions as the clone splits into distinct paths.

### Envelope structural evolution optimizes DH270 clone affinity maturation

**Fig. 7:**
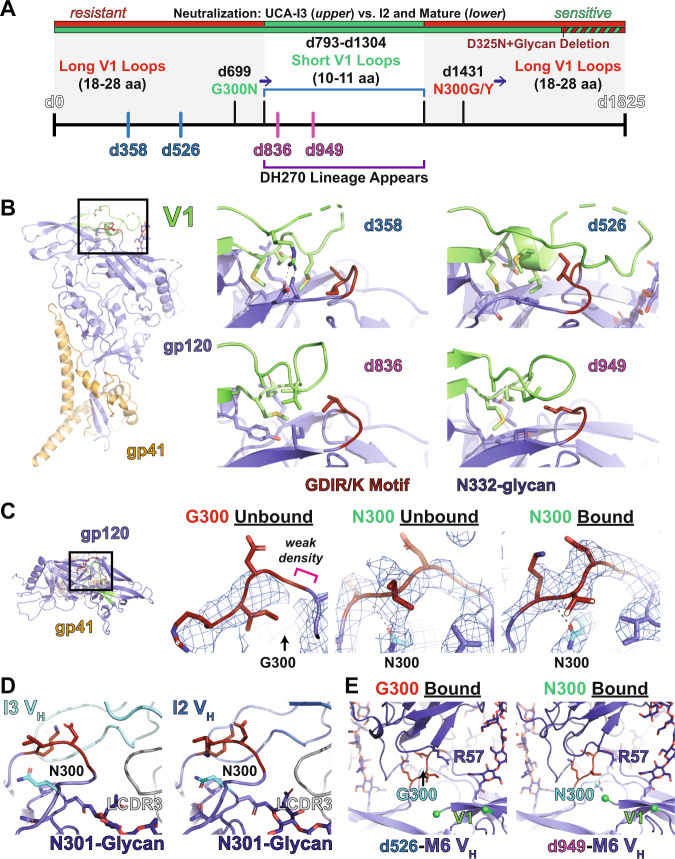
*Envelope-DH270 clone structural coevolution*. **A** Timeline of antibody and Env coevolution. The upper boxes indicate whether Envs at each time point are sensitive (green) or resistant to early intermediates (upper) and later intermediates/mature (lower) clonal members. Periods of infection at which long V1 loops are dominant are shown in grey with key mutation time points are highlighted along the timeline axis. Env structures were determined for sequences isolated at days 358, 526, 836, and 949 post infection highlighted on the timeline axis with period at which the D270 clone appeared highlighted. **B** (left) Side view of a single gp120/gp41 protomer highlighting the V1 and V3 GDIR/K motif. (right) V1 loop structures of each Env isolate. **C** (left) Top view of a single gp120/gp41 protomer highlighting the V1 and V3 GDIR/K motif. Maps and structure fits for the unliganded d358 (middle left), unliganded d949 (middle right), and I3 bound d949 (right) structures. **D** The I3 (left) and I2 (right) bound structures showing similarity in epitope conformation. **E** The DH270.6 bound d526 structure (left) and DH270.6 bound d949 structure (right) showing similarity in epitope conformation.

The evolution of HIV-1 neutralization breadth in the DH270 clone occurred in the backdrop of co-evolution with the virus, with key changes in the Env protein driving antibody maturation, and vice versa^20^. We first examined how the DH270 epitope evolved during infection in the CH848 HIV-1 infected individual (Supplemental Fig. 11). The first DH270 clone antibodies appeared between 793- and 1304-days post infection coinciding with a reduction in Env V1 loop length by 18-19 amino acids and a G300N substitution in Env at a location proximal to the DH270 epitope that appeared at day 699 post-infection (Fig. 7A). The early I5 and I3 intermediates recognize only the Envs that harbor these shorter V1 loops and the G300N mutation. Consistent with Env selection driven by the earlier DH270 clone antibodies I5 and I3 (Supplemental Fig. 12A), autologous viruses with longer V1 loops and G300 re-emerge 1304-1431 days post-infection. The ability of the DH270 clone to neutralize viruses with both longer V1 loop lengths and G300 appears at the I2 intermediate (Fig. 7A, Supplemental Fig. 12). Recognition of longer V1 loops and residue 300 mutations in CH848 quasispecies is also mirrored in DH270 antibody recognition of heterologous viruses with these features, contributing to the enhanced breadth of I2 and DH270.6 as compared to I5 and I3^20^.

Based on these two early intermediate resistance features, we selected Envs from days 358 and 526, having both G300 and long hypervariable V1 loops of 18 and 28 amino acids, respectively, and Envs from days 836 and 949, having both N300 and short hypervariable V1 loops of 11 amino acids, to identify structural hurdles to DH270 antibody interaction. We first examined binding of the clone antibodies to each Env SOSIP ectodomain by ELISA (Supplemental Fig. 13). Consistent with previous observations, the UCA displayed only binding to the d949 construct with I5 binding the d949 and the d836 constructs. While I3 displayed binding to the d358 construct, I4 did not. Consistent with differences in the early maturation of these clones, the mature DH270.2 and DH270.3 antibodies bound weaker to the d358 construct compared to I2, I1, and DH270.4-6. Neither DH270.2 nor DH270.3 bound the d526 construct while measurable binding to I2, I1 and DH270.4-6 was observed, though the interaction was weaker than to the other three constructs. Despite poor sequence similarity, examination of the V1 loop regions of each unliganded Env structure demonstrated a structurally conserved hydrophobic core and variable hydrogen bonding and/or salt bridge formation that ensures coupling of the V1 loop to the V3 GDIR/K motif. The d358 Env V1 displayed considerable disorder in a portion of the loop with flanking hydrophobic and salt bridge interactions ensuring the loop remained closed over the V3 GDIR/K motif (Fig. 7B and Supplemental Fig. 14A). The d526 Env contained a similar hydrophobic core and displayed more order despite its longer length. This was due to direct V1 loop interaction with the nearby N332-glycan (Fig. 7B and Supplemental Fig. 14B). The d836 and d949 Env V1 were markedly shorter but still retain the hydrophobic core and V3 GDIR/K motif coupled position of the earlier viruses (Fig. 7B and Supplemental Fig. 15). The d836 loop displayed a kink in the loop near the V3 GDIR/K motif leading to somewhat greater exposure of the motif (Fig. 7B and Supplemental Fig. 15C). Together, these results indicate a shared role of the V1 for protecting the neutralization sensitive V3 GDIR/K motif that is mediated by a conserved hydrophobic core.

We next examined the effect on the DH270 epitope of the Env G300N substitution that is associated with clone initiation. The V3 GDIR/K motif is proximal to position 300, forming a paired, broken beta sheet secondary structure. Visual inspection of the V3 GDIR/K motif in the G300-containing d358 and d526 Env maps suggested greater disorder in the broken sheet structure compared to that of the N300-containing d836 and d949 Env maps (Fig. 7C). N300 formed a hydrogen bonding contact with the I326 backbone of the V3 GDIK motif in the unbound state. This interaction is retained in the bound state structures for the early intermediates with little change in the V3 GDIK motif conformation (Fig. 7C). These results suggest the G300N stabilized the DH270 epitope enabling the UCA and early intermediates to bind and neutralize the virus. Mutations at residue N300 begin to occur at 1431 days post infection (Supplemental Fig. 11) at which point glycine and tyrosine variants co-circulate with asparagine variants. This occurs around the time that the LCDR3 mutations proximal to Env residue 300 occur in I2, DH270.1, and DH270.3, suggesting the evolving virus selected for these mutations in the clone. Comparison of the Env-bound I3 and I2 structures indicated little overall change in their epitope configuration (Fig. 7D). Additionally, structures of DH270.6 bound to d526 Env containing G300 and d949 Env containing N300 show few changes in the overall epitope conformation (Fig. 7E and Supplemental Fig. 14B). As the principal effect of the LCDR3 mutations was rigidification of the LCDR3 and closer contact with the N301-glycan base, with little change to the bound state configuration in the unliganded or bound states, these mutants likely enhanced the interaction by compensating for increased epitope flexibility. Additional coevolving Env sites outside the V1 loop and position 300 included positions 325 to 327 in the ^324^GDIR/K^327^ motif and position 328 (Supplemental Fig. 11 right). Mutations also occurred that eliminated the N301, N332, and N442 glycan sequons beginning at 1304 days post-infection. Autologous viruses that were resistant to the mature DH270.4 were predominantly enriched for the D325N substitution, but also had loss of glycans at 301, 332, and/or 442. A clear structural impact for D325N was not observed while the close contacts in all structures with the glycans suggest their loss would indeed have major impacts on DH270 antibody binding. Together, these results show that virus evolution initiated DH270 clonal evolution by stabilizing the GDIR/K epitope through G300N and removing the long V1 loop protection of the conserved V3 loop V3 GDIR/K motif. The return of these protective features likely spurred the maturation of the clone toward stabilization of the paratope to combat enhanced flexibility, resulting in greater neutralization breadth.

## Discussion

Here we have studied a series of cryo-EM structures and correlated them with antibody affinity outcome in a prototype V3-glycan HIV-1 broadly neutralizing antibody B cell clone. We demonstrated the early G57R and S27Y mutations that were required for acquisition of heterologous breadth by the I5 intermediate antibody altered antibody orientation relative to Env, and this shift in antibody position likely affected downstream antibody development. The next steps in the affinity maturation of DH270.6 involved optimizing interactions with the N332-glycan with both the I3 and I4 intermediates acquiring mutations around the N332-glycan interaction, albeit targeting different arms of the branched glycan. Maturation of I4 to DH270.2 involved acquiring mutations proximal to the N332-glycan D1 arm like those in I3, suggesting that engaging the D1 arm rather than the D2/3 arm was a better solution to optimizing N332-glycan interaction. If DH270.2 shows that I4 selection was not ideal, DH270.3 shows the trouble with moving on from a critical juncture too soon. Post-I3, development to I2 and DH270.1 along with DH270.3 all showed a considerable number of mutations in the LDCR3 and adjacent residues with I2 acquiring the greatest neutralization breadth and potency. Paratope modification downstream from the I2 intermediate appeared further from the primary epitope and likely acted to fine-tune affinity, breadth, and potency. These results showed staged, site-specific development determined the fate of downstream DH270 clonal affinity maturation.

A recent study of a broadly neutralizing influenza hemagglutinin targeting antibody B cell clone suggested affinity maturation involves developing a diverse pool of potential solutions from which further optimization could induce a broad response^4^. Consistent with that study, here we found that differing mutations solved similar problems presented by the pathogen. However, these solutions were not necessarily equally effective, and the developing clone appeared to evolve toward optimal solutions to gain affinity as evidenced by clade mutation similarity. What supports this antibody “pluripotency” hypothesis developed based on an influenza bnAb^4^ creates a vexing problem for vaccine design. As specific endpoints in clones are desired for their breadth and potency properties, careful steering of developing responses towards these endpoints and away from less productive solutions for neutralization breadth will likely be required.

Our results indicated that solutions to epitope-paratope pairing issues, rather than occurring together, resulted in clade diversification. Seen from the perspective of the entire DH270 clone, failure to acquire an optimal solution limits further affinity maturation as was the case for clonal members DH270.1 and DH270.3. Thus, failing to acquire optimal solutions for antigen recognition at specific problem sites early in bnAb lineage maturation may hinder favorable development neutralization breadth in later lineage members. In the context of the DH270 clone, vaccination that fails to induce the selection of the I5 G57R and S27Y mutations may limit the acquisition of the I3 R98T and L48Y mutations. Further, our results demonstrated how the DH270 clone took different paths to arrive at solutions that differed substantially in HIV-1 neutralization breadth. Knowing which epitope sites may be involved in selecting mutations with better neutralization breadth will better equip us to precisely select immunogen modifications that would produce a positive differential affinity gain favoring the desired mutation(s). Unlike I3, which contained two critical mutations likely to result in affinity gain individually, I2 acquired multiple LCDR3 mutations that worked together to modify interaction with the Env. The need to induce multiple mutations that may do little to improve affinity individually will likely require thinking beyond immunogen properties to issues related to adjuvant, vaccination timing, and the number and identity of boosting immunogens.

While our study focuses on the maturation pathway of a single V3-glycan targeting bnAb clone, several similarities exist between diverse V3-glycan targeting antibodies. While specific details of their maturation paths may differ, antibodies targeting this site have to circumvent similar structural barriers to arrive at optimal solutions to acquire heterologous neutralization breadth. Key among these structural barriers include the V1 loop that the developing antibodies need to circumvent to access the conserved V3 GDIR/K motif, and the epitope glycans, primarily the N332-glycan. Indeed, the ability to simultaneously recognize these two structural motifs defines a V3-glycan bnAb or a precursor. The DH270 clone demonstrates that these epitope sites are engaged early in the clonal development with the DH270 UCA already in an orientation that embraces the N332-glycan, with the next step in the development being the acquisition of the V_H_ G57R mutation to engage the V3 GDIK motif. Therefore, antibody orientation bracketed within the V3 GDIR/K motif and the N332-glycan, even during early stages of clonal development where no or little neutralization breadth may be observed, is a key structural signature of a V3-glycan antibody that may develop to acquire heterologous neutralization breadth. This contrasts with other spatially proximal HIV-1 Env apex targeting autologous antibodies that engage the V3 GDIR/K motif but not the N332-glycan^31–33^.

Emerging evidence suggests an effective HIV-1 vaccine will require inducing a polyclonal bnAb response targeting multiple Env sites of vulnerability^26^. One path to realizing this difficult task is to design immunogens that can select for known bnAb mutations in germline precursor B cells. Mapping antibody bnAb development in this study provides a roadmap to such precision, clone-based immunogen development. By isolating distinct structural elements at the antibody–antigen interface, sites of gain can be established, and immunogens can be developed to sequentially solve structural problems associated with each site individually. By shifting focus to specific, manageable steps, rational affinity design can proceed through a series of well-defined targets rather than attempting to implement a speculative set of final immunogen designs lacking clear data.

Statistical inference plays a key role in our examination of DH270 lineage antibody and CH848 HIV-1 Env co-evolution. Antibody clonal tree reconstruction uses maximum likelihood-based statistical inference to estimate the antibody sequences of internal nodes representing ancestral states of the B cell clone and the accuracy of this method is limited by the available sequence data sampled for the clone and the evolutionary model used. As such, the clonal tree for DH270 clone used here and in other studies^8,34^, represents the best estimate given the default parameters of the Cloanalyst program^35^ and the sequence data from the 6 paired heavy and light chains of the DH270 clone recovered by antigen-specific single B cell sorting. Our longitudinal Env sequence analysis too depends on statistical inference and the relationship between clonal lineage member virus neutralization and the likelihood that a particular Env feature is selected for antibody changes that enhance breath. Our reporting on the structural effects of DH270 lineage antibody mutations and specific Env mutation effects in the context of co-evolution therefore depend on the veracity of these inferences. Further, the exact Env sequence that initiated the lineage and the number of different sequences that drove DH270 clonal lineage development is not known.

While changes in the virus and the antibody clone occurred concurrently during natural co-evolution, most of our structural observations rely on the use of a single Env, which is a soluble SOSIP trimer derived from a virus isolated from the CH848 individual at day 949 after transmission. Although this precluded a faithful structural replica of the co-evolution process, it allowed us to keep the dataset manageable while still gleaning meaningful insights into the co-evolution by focusing on specific Env residue changes that were determinants in the emergence of resistance and in driving antibody evolution. Our strategy allowed us to study the effects of antibody residue changes without being confounded by simultaneous changes in Env. Finally, we added further insights into the co-evolution by studying select Envs with differential neutralization properties and structural barriers to antibody engagement that arose at different time-points.

## Methods

### Recombinant antibody production

Expi293 cells (ThermoFisher Cat No. A14527) were diluted to a final volume of 0.5 L at a concentration of 2.5 × 10^6^ cells mL^-1^ in Expi293 media. Four hundred micrograms of heavy chain and light chain plasmid were complexed with Expifectamine (ThermoFisher Cat No. A14526) and added to the Expi293 cells. On day five, cells were cleared from transfection cell culture media by centrifugation and 0.8 μM filtration. The supernatant containing recombinant antibody was incubated with protein A resin (ThermoFisher) overnight at 4 °C. The protein A resin was collected by centrifugation and culture supernatant was removed. The resin was washed with 25 mL of phosphate-buffered saline (PBS) containing a total of 340 mM NaCl. A total of 30 mL of 10 mM glycine pH 2.4, 150 mM NaCl were used to elute the antibody off the protein A resin. The pH of the eluted antibody solution was increased to approximately 7 by the addition of 1M Tris pH 8.0. The antibody solution was buffer exchanged into PBS with successive rounds of centrifugation, filtered, and stored at −80 °C.

### Recombinant HIV-1 envelope SOSIP gp140 production

CH848 SOSIP gp140 envelope production was performed with Freestyle293 cells (ThermoFisher Cat No. R79007). On the day of transfection, Freestyle293 was diluted to 1.25 × 10^6^ cells/mL with fresh Freestyle293 media up to 1 L total volume. The cells were co-transfected with 650 μg of SOSIP-expressing plasmid DNA and 150 μg of furin-expressing plasmid DNA complexed with 293Fectin (ThermoFisher Cat No. 12347019). On day 6 cell culture supernatants were harvested by centrifugation of the cell culture for 30 min at 3500 rpm. The cell-free supernatant was filtered through a 0.8 μm filter and concentrated to less than 100 mL with a single-use tangential flow filtration cassette and 0.8 μm filtered again. Trimeric Env protein was purified with monoclonal antibody PGT145 affinity chromatography. PGT145-coupled resin was packed into Tricorn column (GE Healthcare) and stored in PBS supplemented with 0.05% sodium azide. Cell-free supernatant was applied to the column at 2 mL/min using an AKTA Pure (GE Healthcare), washed, and protein was eluted off of the column with 3M MgCl_2_. The eluate was immediately diluted in 10 mM Tris pH 8, 0.2 μm filtered, and concentrated down to 2 mL for size exclusion chromatography. Size exclusion chromatography was performed with a Superose 6 16/600 column (GE Healthcare) in 10 mM Tris pH 8, 500 mM NaCl. Fractions containing trimeric HIV-1 Env protein were pooled together, sterile-filtered, snap-frozen, and stored at −80 °C.

### Surface plasmon resonance

The SPR binding curves of the DH270 lineages Fabs against CH848 DS SOSIP Trimer were obtained using either a Biacore S200 instrument (Cytiva) in HBS-N 1× running buffer or a Biacore 3000 instrument (Cytiva) in PBS 1× pH7.4. For DH270 Fabs I5.6, I3.6, I2.6, and DH270.6 affinity measurements, biotinylated CH848 DS SOSIP Trimer was immobilized onto a CM3 sensor chip via streptavidin to a level of 280–290RU. Using the single cycle injection type, six sequential injections of the Fabs diluted from 50 to 1500 nM were injected over the immobilized SOSIP Trimer at 50 µL/min for 120 s per concentration followed by a dissociation period of 600 s. The Fabs were regenerated with a 20s pulse of 25 mM NaOH at 50 µL/min. Results were analyzed using the Biacore S200 Evaluation Software (Cytiva). A blank streptavidin surface as well as buffer binding were used for double reference subtraction to account for non-specific antibody binding and signal drift. Curve fitting analyses were performed using the 1:1 Langmuir model with a local Rmax. For DH270UCA3 Fab, biotinylated CH848 DS SOSIP Trimer was immobilized onto a CM5 sensor via streptavidin to a level of 290–300RU. DH270UCA3 Fab was diluted from 500 to 3000 nM and each concentration was injected over the SOSIP Trimer surface for 300 s using the K-inject injection type at 50 µL/min. A 600s dissociation period was followed by surface regeneration with a 20 s pulse of 50 mM NaOH. Results were analyzed using the BIA evaluation software (Cytiva). A blank streptavidin surface and buffer binding were used for double reference subtraction. Curve fitting analysis of DH270UCA3 Fab was performed using the 1:1 Langmuir model with a local Rmax. The reported binding curves for all Fabs are representative of one data set.

### Cryo-EM sample preparation

The CH848 SOSIP trimer complexes were prepared using a stock solution of 2 mg/ml trimer incubated with a six-fold molar excess of Fab. To prevent the interaction of the trimer complexes with the air-water interface during vitrification, the samples were incubated in 0.085 mM *n*-dodecyl β-D-maltoside (DDM). Samples were applied to plasma-cleaned QUANTIFOIL holey carbon grids (EMS, R1.2/1.3 Cu 300 mesh) followed by a 30 s adsorption period and blotting with filter paper. The grid was then plunge frozen in liquid ethane using an EM GP2 plunge freezer (Leica, 90-95% relative humidity).

### Cryo-EM data collection

Cryo-EM imaging was performed on a FEI Titan Krios microscope (Thermo Fisher Scientific) operated at 300 kV. Data collection images were acquired with a Falcon 3EC or K3 Direct Electron Detector operated in counting mode with a calibrated physical pixel size of 1.08 Å with a defocus range between −1.0 and −3.5 µm using the EPU software (Thermo Fisher Scientific). No energy filter or C_s_ corrector was installed on the microscope. The dose rate used was ∼0.8 e^-^/Å^2^ s to ensure operation in the linear range of the detector. The total exposure time was 60 s, and intermediate frames were recorded every 2 s giving an accumulated dose of ∼42 e^-^/Å^2^ and a total of 30 frames per image.

### Data processing

Cryo-EM image quality was monitored on-the-fly during data collection using automated processing routines. For datasets DH270.I3, DH270.I2, DH270.6, DH270.I4, and DH270.2, data processing was performed within cryoSPARC^36^ including particle picking, multiple rounds of 2D classification, ab initio reconstruction, homogeneous map refinement, and non-uniform map refinement. The rest of the datasets were further processed outside of cryoSPARC to improve map quality as described next. Movie frame alignment was carried out using UNBLUR^37^, and CTF determination using CTFFIND4^38^. Particles were picked automatically using a Gaussian disk of 80 Å in radius as the search template. All the picked particles were extracted with box size of 384 pixels and down scaled to 192 pixels (binning 2) and subjected to 8 rounds of refinement in cisTEM^39^, using an ab-initio model generated with cryoSPARC^36^. The distribution of scores assigned to each particle by cisTEM showed a clear bi-modal distribution and only particles in the group containing the higher scores were selected for further processing. The clean subset of particles were re-extracted without binning and subjected to 10 iterations of local refinement followed by 5 additional iterations using a shape mask generated with EMAN2^40^. Per-particle CTF refinement was then conducted until no further improvement in the FSC curve was observed. At this point, particle frames were re-extracted from the raw data and subjected to per-particle motion correction using all movie frames and applying a data-driven dose weighting scheme^41^. The new locally aligned particles were exported to cryoSPARC to conduct an additional round of homogeneous refinement and non-uniform refinement.

For elbow angle analysis with DH270.I3, I4, and DH270.5, local focus refinement with the clean particles after symmetry expansion was done in cryoSPARC. Then, the particles together with corresponding refinement parameters were then exported into RELION 3.1^42^ to perform 3D classification without alignment using T=25.

### Cryo-EM structure fitting

Structures of each antibody were prepared using Modeller^43^ and available Fab crystal structures^20,30^. The SOSIP Env structures were prepared using modeler and the DH270.6 bound CH848 day 949 trimer (PDB ID 6UM6 chains A and B)^21^. Coordinates for VRC01 from PDB ID 3NGB^44^ (chains H and L). Structure fitting of the cryo-EM maps was performed in ChimeraX^45^ using the Isolde application^46^. Structure fit and map quality statistics were determined using MolProbity^47^ and EMRinger^48^, respectively. Glycans were fit using a combination of the sharpened and gaussian filtered maps (1.0–1.5 standard deviation). Structure and map analyses were performed using a combination of PyMol^49^ and ChimeraX with theta and phi angles calculated using VMD^50^. Electrostatic potential maps were generated in PyMol.

### ELISA

Antibody binding to SOSIP proteins was tested using 384-well ELISA plates were coated with 2 mcg/ml of antibody in 0.1 M sodium bicarbonate overnight at 4 °C. Plates were washed with PBS/0.05% Tween-20 and blocked with assay diluent (PBS containing 4% (w/v) whey protein/15% Normal Goat Serum/0.5% Tween-20/0.05% Sodium Azide) at room temperature for 1 h. Purified SOSIP protein was added in 2-fold serial dilutions starting at 10 mcg/ml for 1 h followed by washing. Binding of SOSIP to the coated antibodies was detected using biotinylated human antibody PGT151 at 0.125 mcg/ml for 1 h. PGT151 was washed and followed by streptavidin-HRP (Thermo Scientific # 21130) 1:30,000 for 1 h. Plates were washed 4 times and developed with TMB substrate (SureBlue Reserve- #5120-0083) followed by 1M HCl to stop the reaction. Finally, the absorbance at 450 nm (OD_450_) was determined with a 384 well plate reader (Molecular Devices, Spectramax 384 plus).

### CH848 longitudinal Env analyses

Env sequences used for signature analysis are from Bonsignori et al.^20^. The webtool AnalyzeAlign at the Los Alamos HIV Database was used for generating sequence logos. Autologous neutralization data are from Bonsignori et al.^20^. measured on 90 autologous Env pseudoviruses including the TF and representative longitudinal Envs sampled between 78 to 1720 days post-infection. While intermediates in the previous study were inferred with 5 clone members instead of 6 as in this current study, intermediates I5, I3, and I2 correspond very closely to IA4, IA2, and IA1, respectively in Bonsignori et al.^20^.

### Reporting summary

Further information on research design is available in the Nature Portfolio Reporting Summary linked to this article.

## Supplementary information

Supplementary Information File

Reporting Summary

## Data Availability

Cryo-EM reconstructions and atomic models generated during this study are available at wwPDB and EMBD (www.rcsb.org; http://emsearch.rutgers.edu) under the following accession codes: Cryo-EM reconstructions and atomic models generated during this study are available at wwPDB and EMBD (www.rcsb.org; http://emsearch.rutgers.edu) under the following accession codes: EMD IDs: EMD-40282, EMD-40283, EMD-40285, EMD-40286, EMD-40287, EMD-40288, EMD-40291, EMD-40290, EMD-40289, EMD-40284, EMD-40280, EMD-40280, EMD-40278, EMD-40277, EMD-40275, EMD-40281, EMD-40279, EMD-40274, EMD-40273 and PDB IDs: 8SAW, 8SAX, 8SAZ, 8SB0, 8SB1, 8SB2, 8SB5, 8SB4, 8SB3, 8SAY, 8SAU, 8SAU, 8SAS, 8SAR, 8SAQ, 8SAV, 8SAT, 8SAN, 8SAL.
