# Supplementary material for: Structural basis for breadth development in the HIV-1 V3-glycan targeting DH270 antibody clonal lineage
Source: Nat Commun. 2023 May 15;14:2782. doi: 10.1038/s41467-023-38108-1 (PMC10184639; doi:10.1038/s41467-023-38108-1)
Supplement: Supplementary file 1 — Supplementary Information File [file 41467_2023_38108_MOESM1_ESM.pdf]

Supplemental Material
Structural basis for breadth development in the HIV-1 V3-glycan targeting DH270 antibody clonal lineage

Rory Henderson, Ye Zhou, Victoria Stalls, Kevin Wiehe, Kevin O. Saunders, Kshitij Wagh, Kara Anasti, Maggie Barr, Robert Parks, S. Munir Alam, Bette Korber, Barton F. Haynes, Alberto Bartesaghi, Priyamvada Acharya

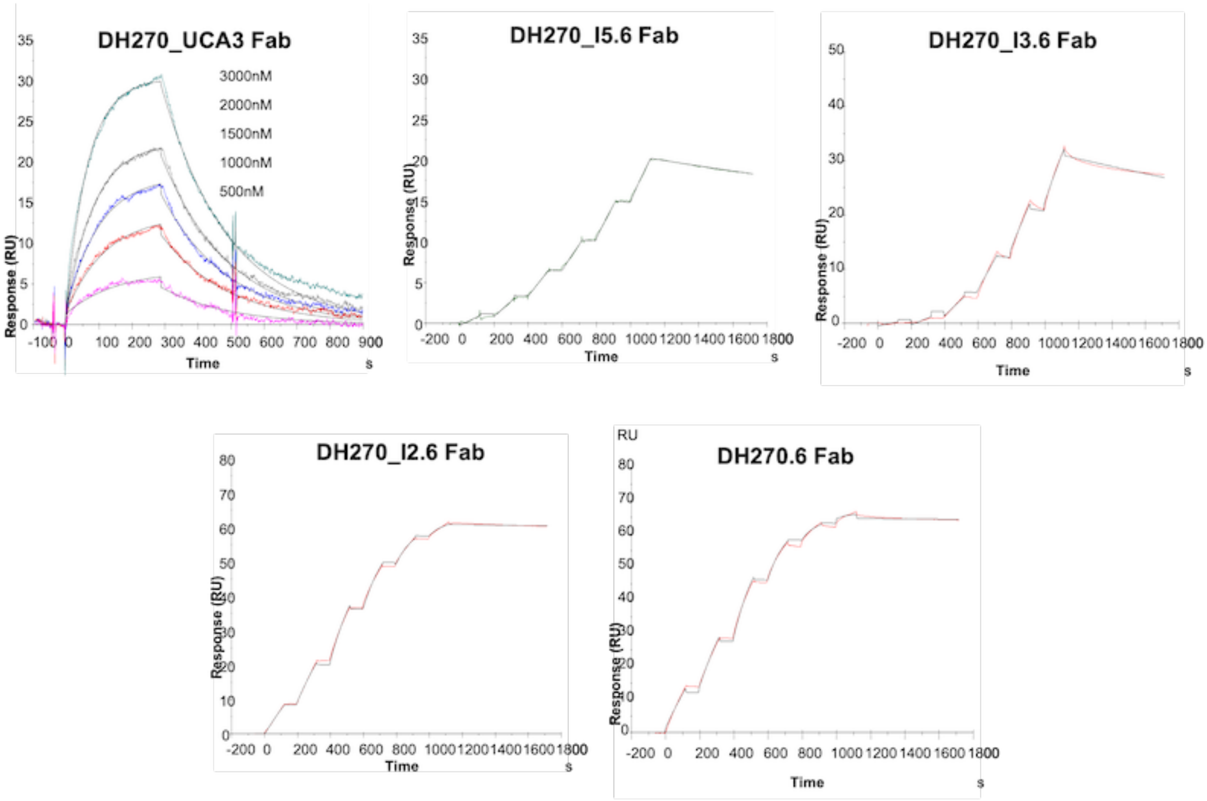

Supplemental Figure 1. Surface plasmon resonance (SPR). Binding curves are overlaid with curves fits used to derive affinities for antibody Fabs leading from the UCA to the mature DH270.6 listed in Figure 1.

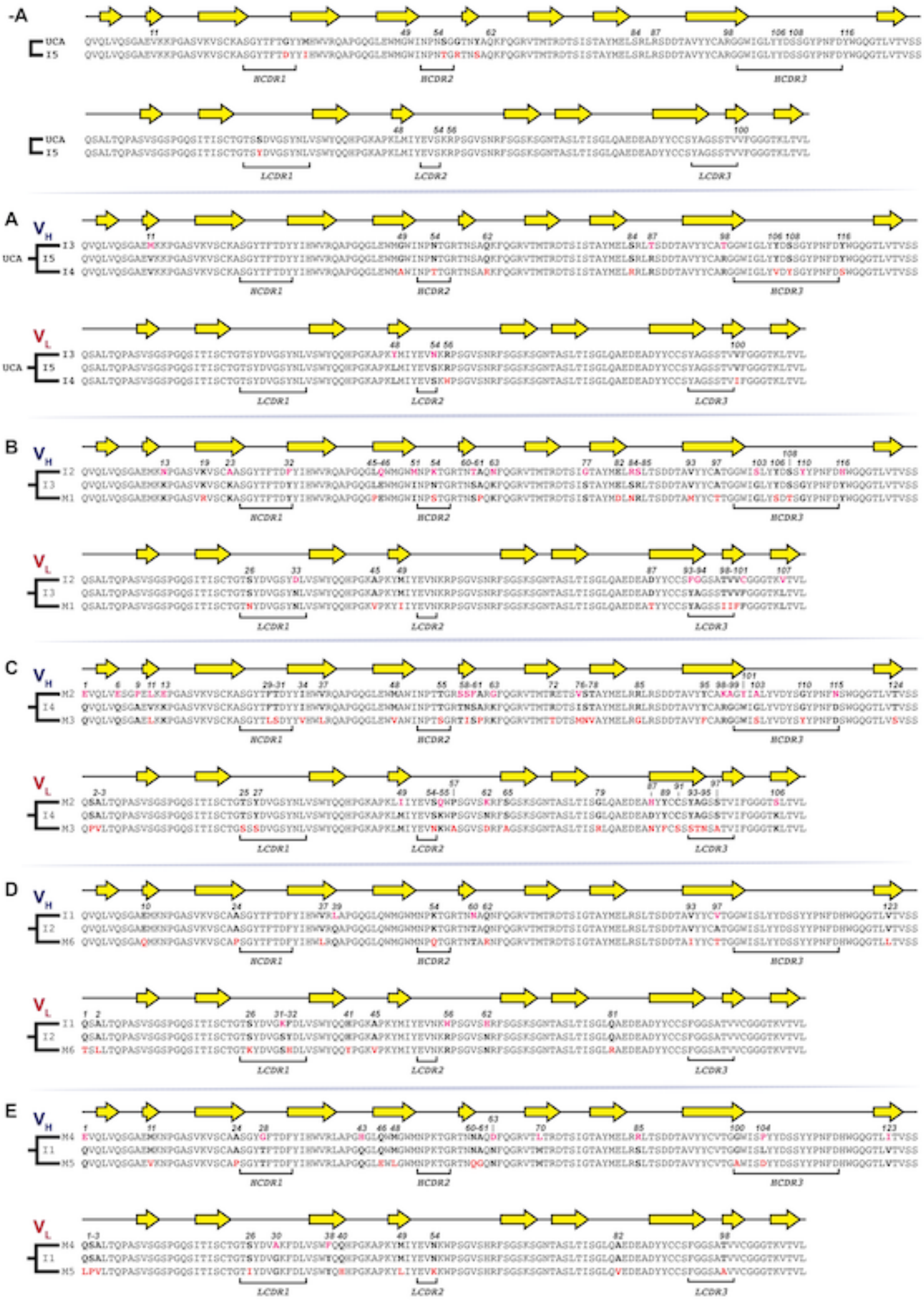

-A
UCA
I5
HCDR1
HCDR2
HCDR3
LCDR1
LCDR2
LCDR3
A
VH
VL
I3
I5
I4
B
I2
M1
C
M2
M3
D
I1
M6
E
M4
M5

Supplemental Figure 2. ***Sequence alignments for the DH270 clonal clone.*** Sequence alignments at each path split with secondary structure and mutations highlighted in bold.

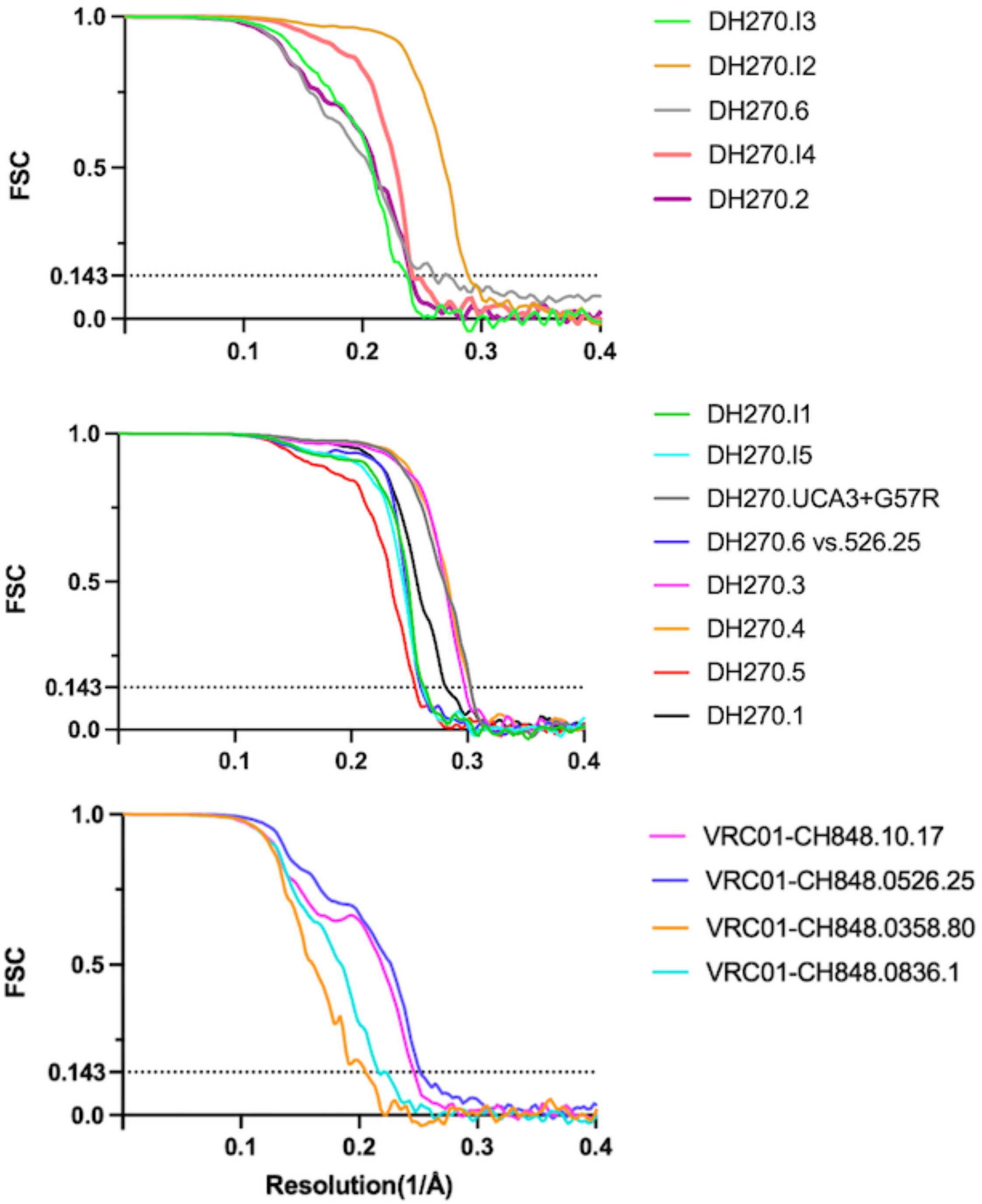

Supplemental Figure 3. ***Fourier shell correlation (FSC) plots for DH270 clonal clone antibody Fab bound maps, set one.*** Gold-standard FSC curves calculated from two independently refined half-maps. The dotted line indicates FSC = 0.143.

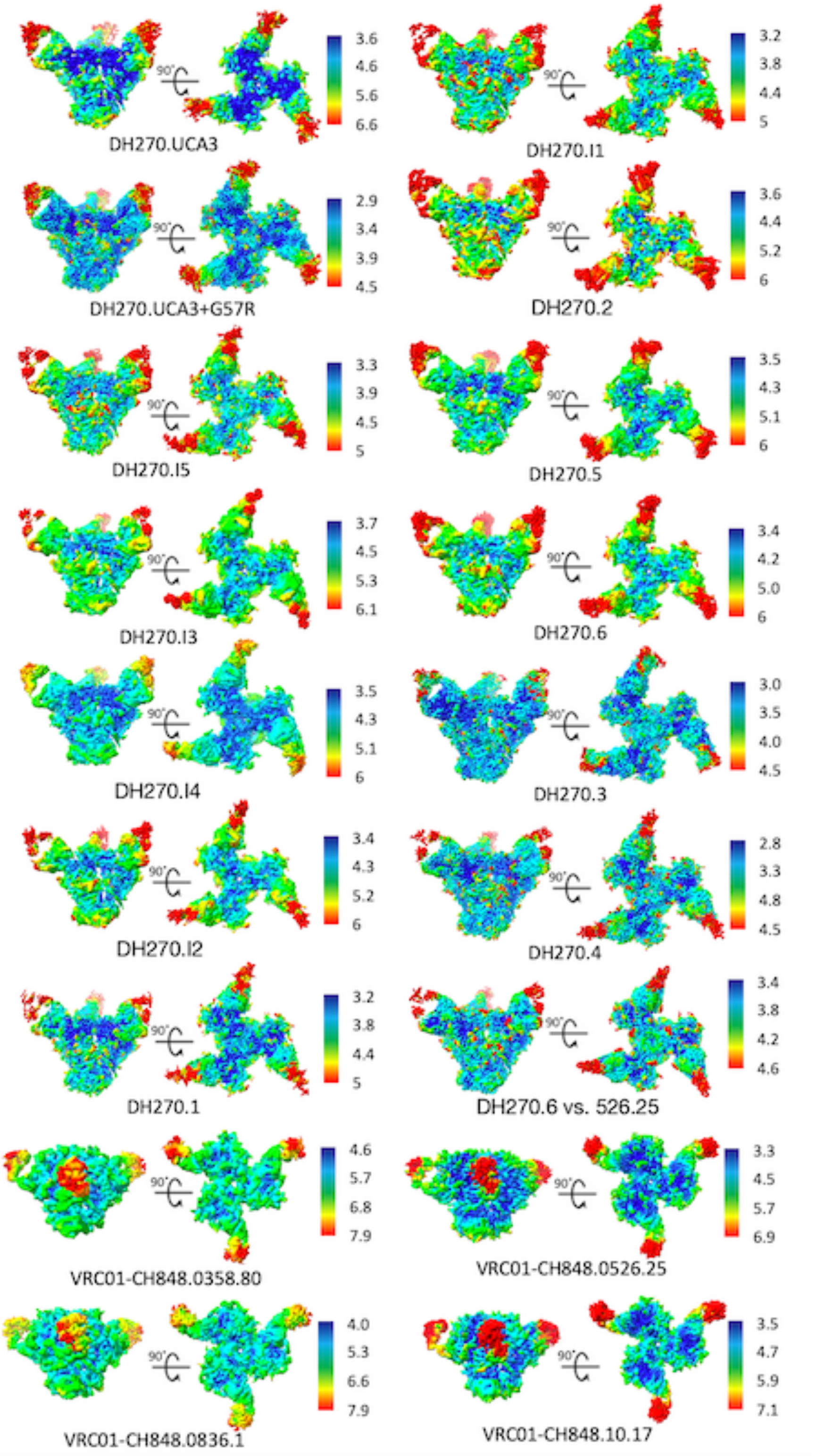

Supplemental Figure 4. ***Fourier shell correlation (FSC) plots for DH270 clonal clone antibody Fab bound maps, set two.*** Gold-standard FSC curves calculated from two independently refined half-maps. The dotted line indicates FSC = 0.143.

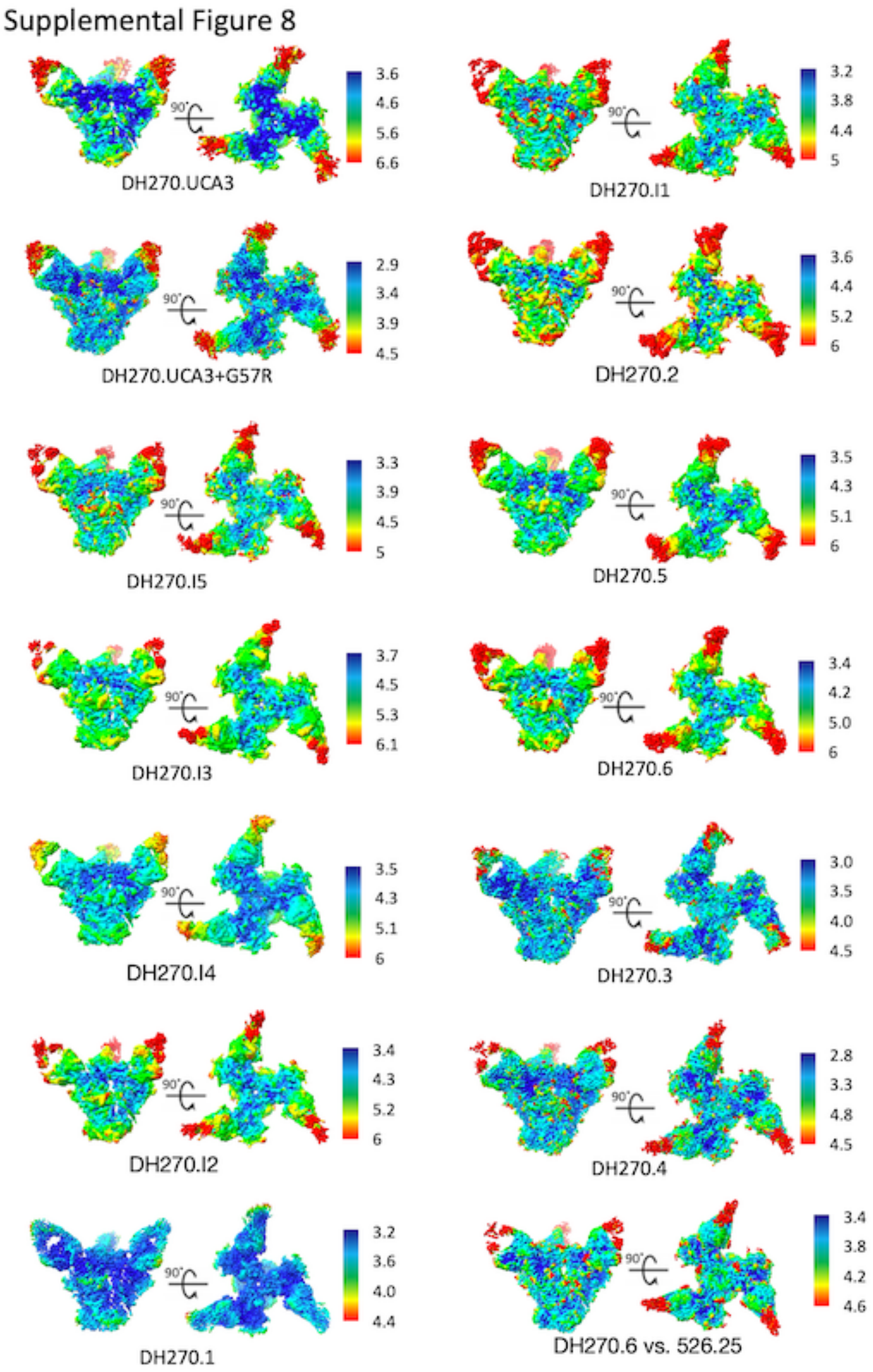

Supplemental Figure 5. ***Local resolution for the DH270 clonal clone antibody Fab bound structures, set two.*** Refined cryo-EM maps for each complex colored by local resolution.

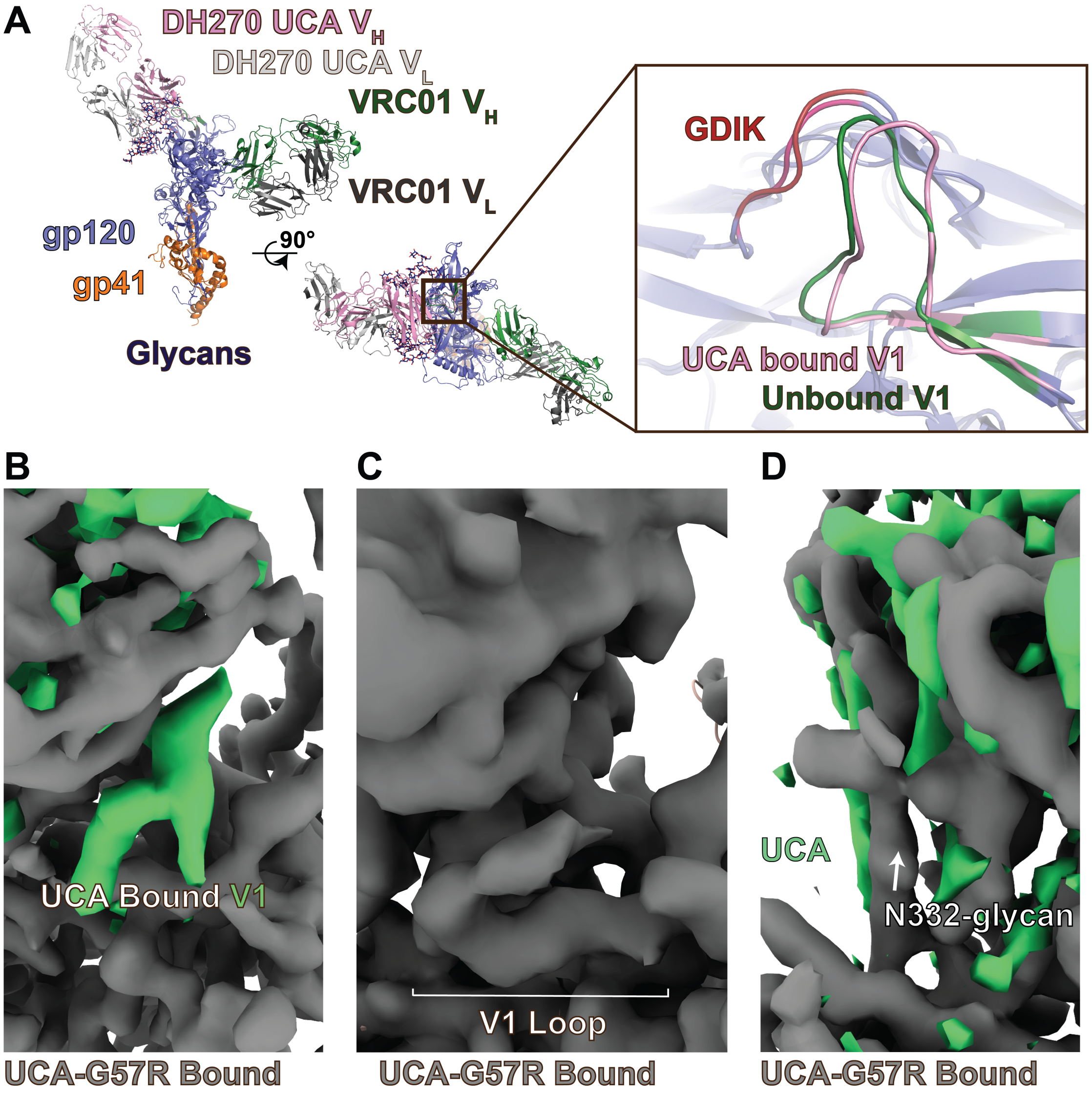

Supplemental Figure 6. ***Changes in V1 loop configuration induced by heavy chain G57R mutation early in DH270 clonal development (UCA to I5).*** **A)** Aligned gp120/gp41 domains of the DH270 UCA and VRC01 bound CH848 d949 structures highlighting similarity between the liganded (UCA bound structure) and unliganded (VRC01 bound structure) epitope V1 loop conformations. **B)** The UCA+G57R map (grey) overlaid with a UCA difference map (green) highlighting V1 loop differences. **C)** A gaussian filtered (standard deviation 1.5) UCA-G57R map highlighting density for the V1 loop. **D)** The UCA+G57R map (grey) overlaid with a UCA difference map (green) highlighting the shift in antibody position.

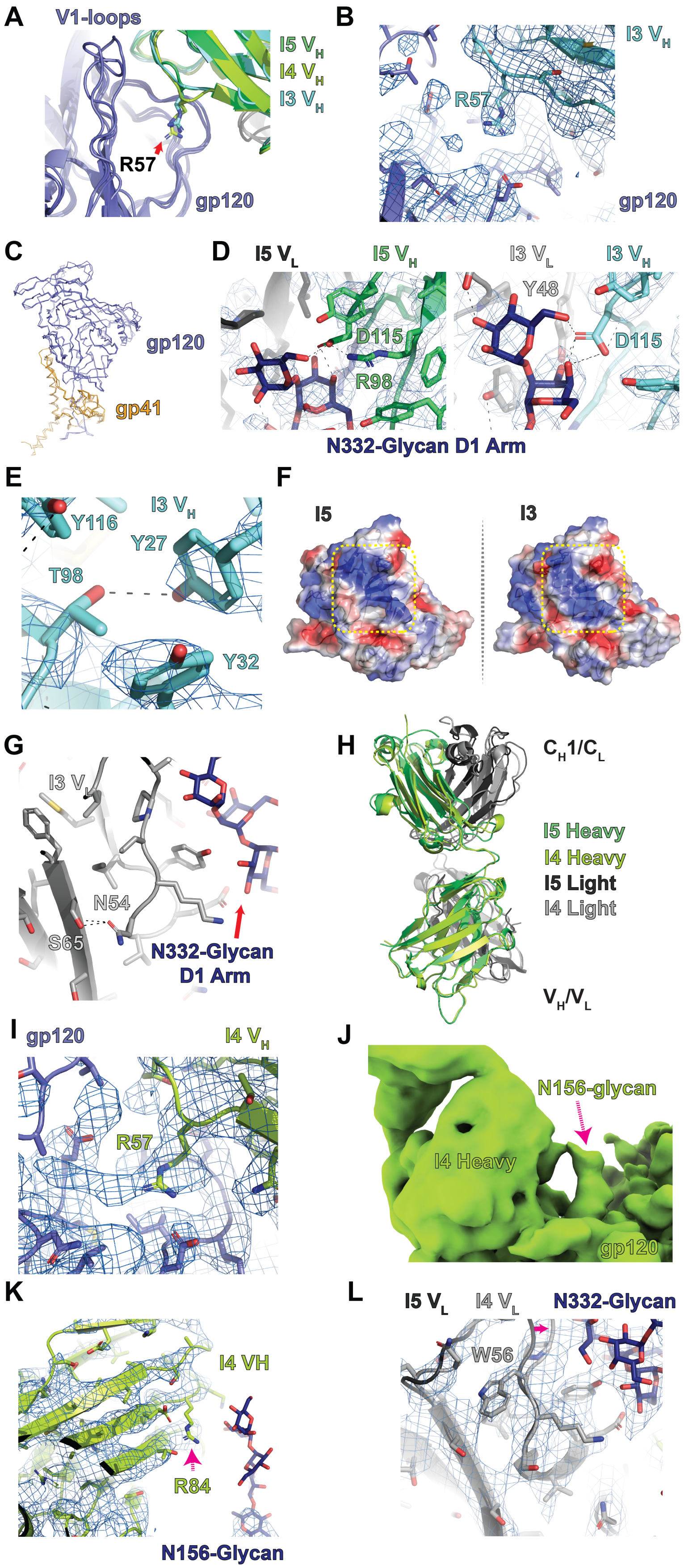
A
V1-loops
I5 VH
I4 VH
I3 VH
R57
gp120
B
I3 VH
R57
gp120
C
gp120
gp41
D
I5 VL
I5 VH
D115
R98
I3 VL
I3 VH
Y48
D115
N332-Glycan D1 Arm
E
Y116
I3 VH
Y27
T98
Y32
F
I5
I3
G
I3 VL
N54
S65
N332-Glycan
D1 Arm
H
CH1/CL
I5 Heavy
I4 Heavy
I5 Light
I4 Light
VH/VL
I
gp120
I4 VH
R57
J
N156-glycan
I4 Heavy
gp120
K
I4 VH
R84
N156-Glycan
L
I5 VL
I4 VL
N332-Glycan
W56

Supplemental Figure 7. ***The I5 path split structures: I3 and I4.*** **A)** gp120 V1 loops displaced by residue R57 of I5, I4, and I3. **B)** Map and fit for the I3 intermediate showing the regions around residue R57. **C)** Alignment of the I5, I4, and I3 bound gp120/gp41 domains. **D)** (left) Map and fit for the I5 intermediate of the regions around residues R98 and D115 showing interactions with the N332-glycan D1 arm. (right) Map and fit for the I3 intermediate of the regions around the R98T and D115 positions showing improved interactions with the N332-glycan D1 arm. **E)** Map and fit of the I3 Fab V_H showing interaction between Y27 and T98. **F)** Comparison of the I5 and I3 intermediate antibody antibody electrostatic potential maps highlighting the effect of the R87T mutation in I3. **G)** Structure of the I3 V_L highlighting hydrogen bonds formed by the N54 mutations. **H)** Aligned I5 and I4 V_H domains showing similar elbow angles. **I)** Map and fit for the I4 intermediate showing the region around residue R57. Map density around the R57 side chain is consistent with a V1 loop. **J)** Gaussian filtered I4 intermediate bound state map showing N156-glycan density. **K)** Map and fit for the I4 intermediate of the region around residue R84. Limited density for the R84 side chain precluded definition of its possible interactions. **L)** Alignment of the I5 and I4 structures with the I4 map showing slight rearrangement in the antibody loop adjacent to the N332 glycan.

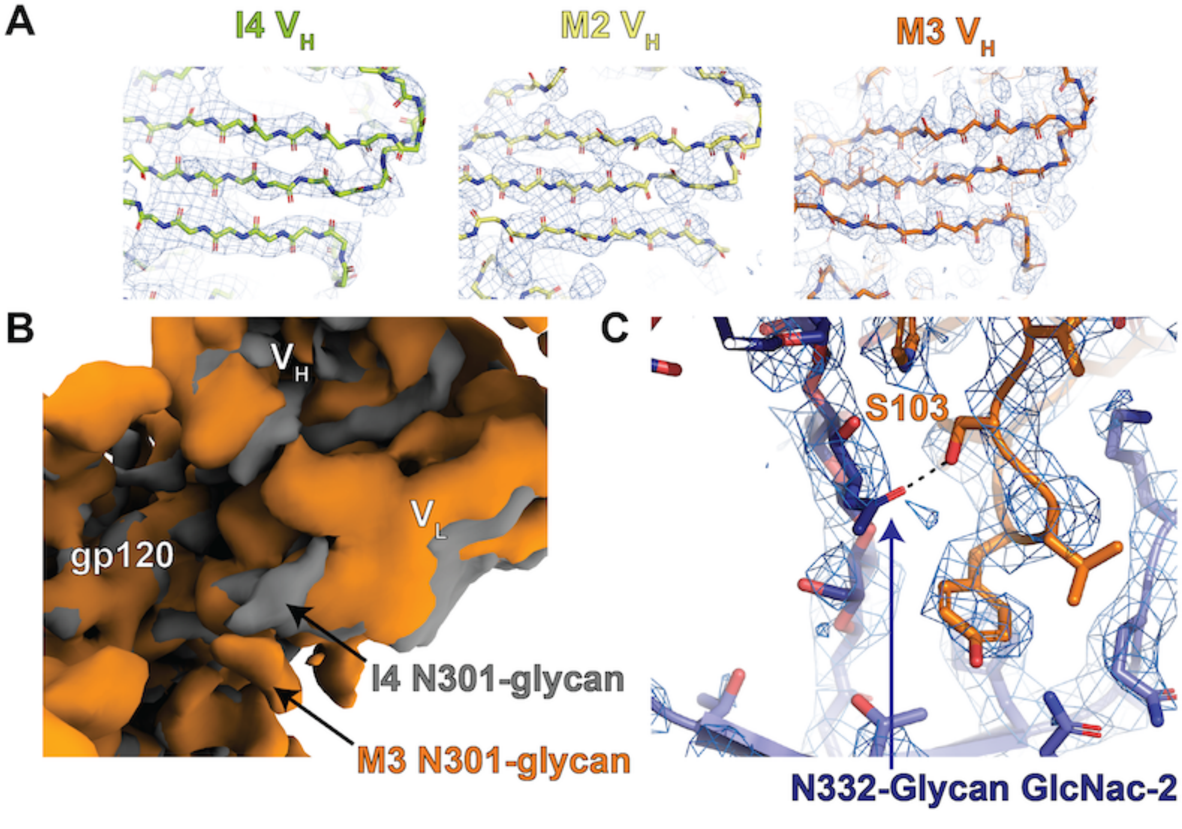

Supplemental Figure 8. ***The I4 path split structures: Mature antibodies DH270.2 and DH270.3.*** **A)** Map and backbone structure fits for the I4, DH270.2 (M2) and DH270.3 (M3) heavy chains V_H domains. **B)** Gaussian filtered map alignments of the I4 and DH270.3 bound maps highlighting the shift in antibody and N301-glycan positions. **C)** Map and fit of the DH270.3 structure in the HCDR3 region highlighting hydrogen bonding between N332-glycan GlcNac-2 and heavy chain residue S103.

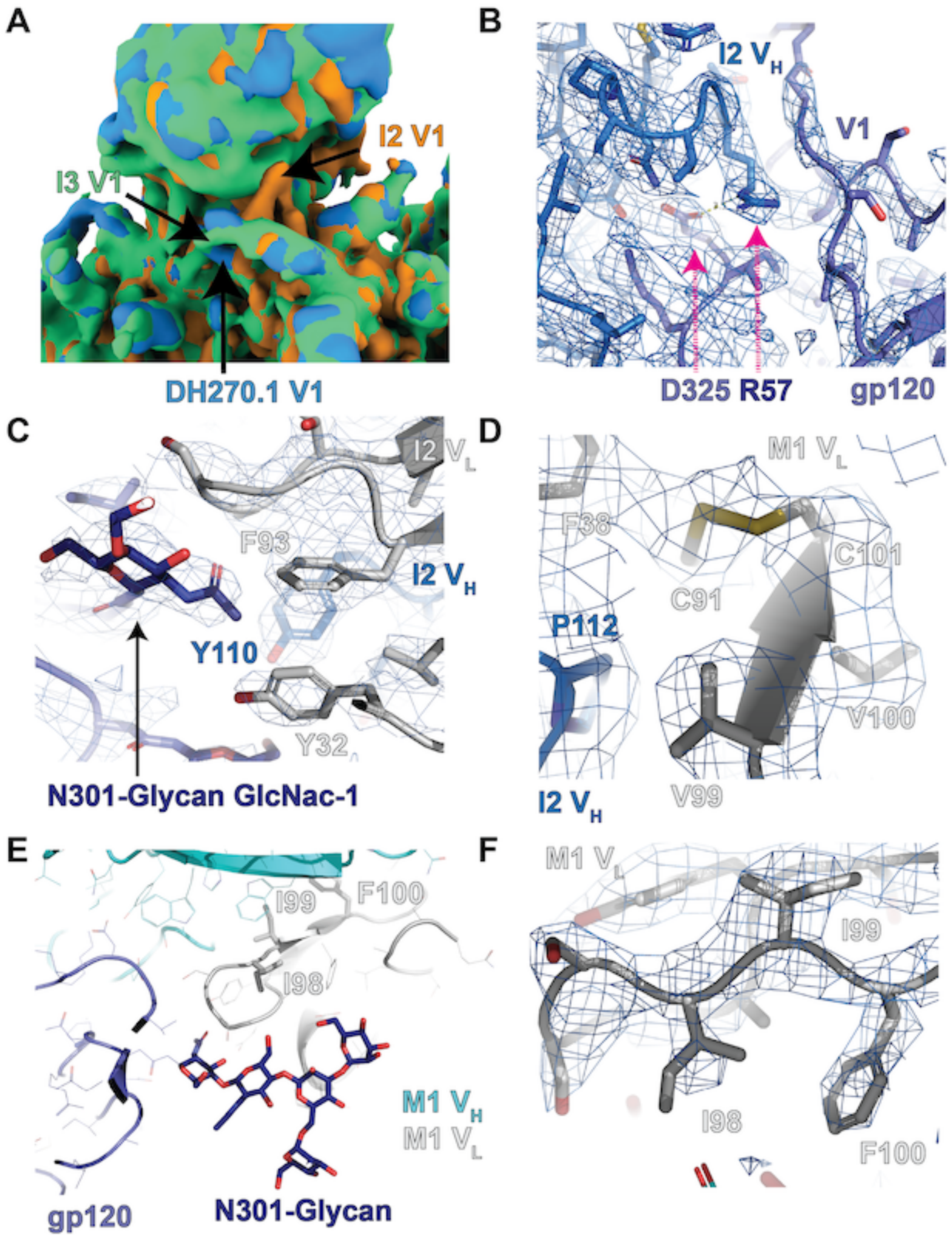

Supplemental Figure 9. ***The I3 path split: Intermediate I2 and mature DH270.1.*** **A)** Gaussian filtered maps of the I3, I2, and DH270.1 (M1) bound maps showing differences in the V1 loop conformations. **B)** Map and fit of the I2 bound structure showing the shift in the I2 R57 side chain toward gp120 residue D325 of the V3 GDIK motif. **C)** Map and fit of the I2 bound structure showing the position of Y110 relative to F93 and the N301-glycan base. **D)** Map and fit of the I2 bound structure showing the newly formed disulfide bond between C91 and the F101C

site. **E)** DH270.1 structure near the LCDR3 mutations. **F)** Map and fit of the DH270.1 bound structures showing the position of the acquired hydrophobic mutations near LCDR3.

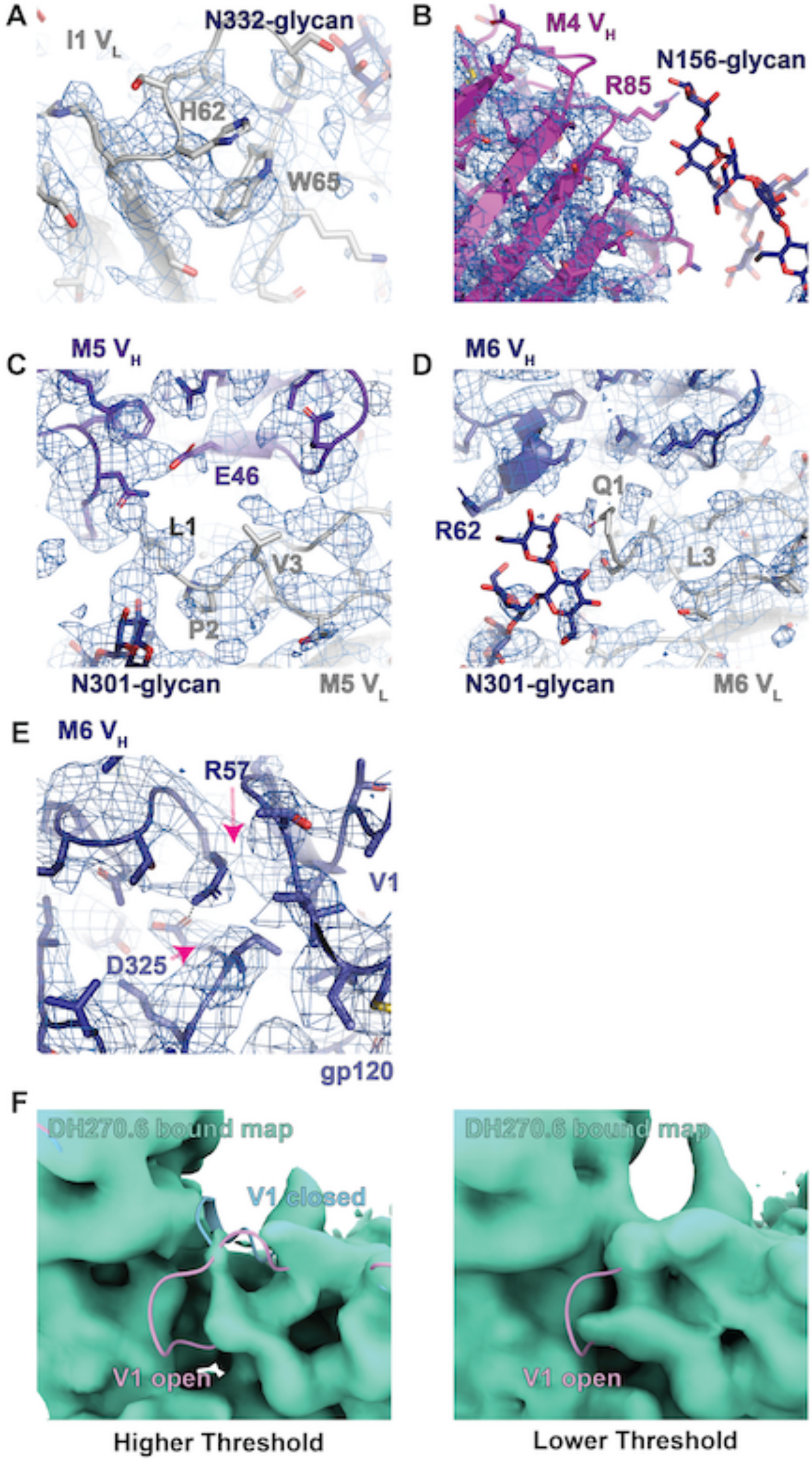

Supplemental Figure 10. ***The I2 path split: Intermediate I1 and mature antibodies DH270.4, DH270.5, and DH270.6.*** **A)** Map and fit of the I1 bound structure at the N332-glycan adjacent light chain loop showing limited density for the mutation sites. **B)** Map and fit of the DH270.4 (M4) bound structure showing that poor map densities at R85 limit inspection of possible N156-glycan interactions. **C)** Map and fit of the DH270.5 (M5) bound structure in the region of the light chain N-terminus highlighting nearby mutations. **D)** Map and fit of the DH270.6 (M6) bound structure in the region of the light chain N-terminus highlighting nearby mutations. **E)** Map and fit of the DH270.6 (M6) bound structure showing the closed gp120 V1 loop and the shifted R57 side chain. **F)** Gaussian filtered map and closed and open V1 loops of the DH270.6 bound structure showing evidence of both a closed and an open state V1.

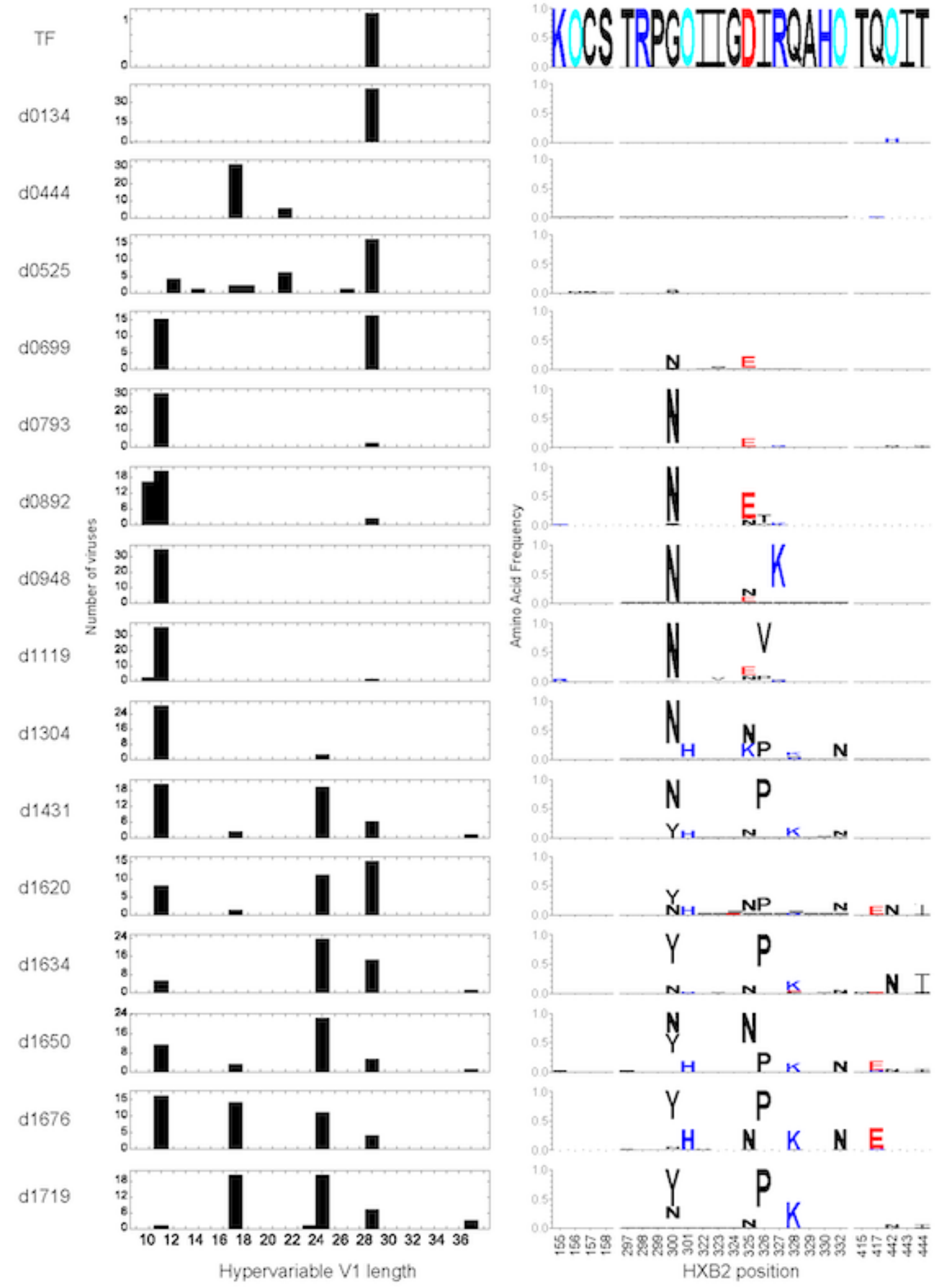

Supplemental Figure 11. ***Longitudinal Env evolution in CH848 at key Env sites.*** The left panels show the distribution of hypervariable V1 loop lengths for longitudinally sampled CH848 Envs at each time point indicated to left of each plot (TF = transmitted founder; d0134 = 134 days post infection). The right panels show frequencies of amino acid and glycan mutations that arise over time at structurally key Env sites. For each plot, the height of the amino acid is proportional to its frequency at that time point, and the TF amino acid, shown only in the top LOGO, is blanked out to accentuate the mutations. Charge-based color-coding of amino acids is used (red: Asp, Glu; blue: His, Arg, Lys; black: all other amino acids), and potential N-linked glycan sites are indicated by "O" and colored cyan.

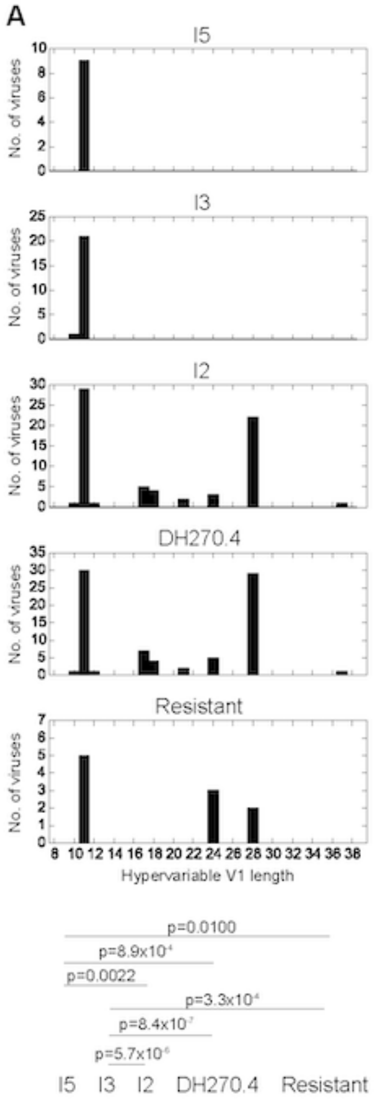

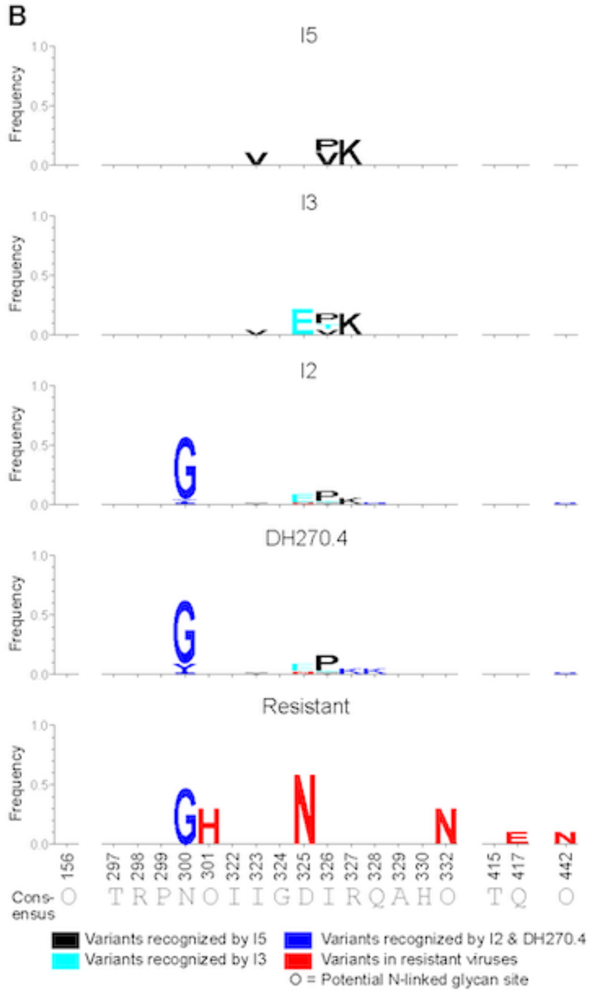

Env Site	Env variants recognized				
	I5	I3	I2	DH270.4	Resistant
300	N	N	NGYS	NGYS	NG
301	O	O	O	O	OH
321	DG	DG	DGA	DGA	D
323	IV	IV	IV	IV	I
325	D	DE	DEN	DEN	DN
326	IPV	IPVT	IPVT	IPVT	I
327	RK	RK	RK	RK	R
328	Q	Q	QK	QK	Q
332	O	O	O	O	ON
417	Q	Q	Q	Q	QE
442	O	O	OH	OH	ON

Supplemental Figure 12. ***Env features of autologous viruses neutralized by intermediate and mature DH270 Abs.*** **A)** Histograms of hypervariable V1 loop lengths for the group of viruses that could be neutralized by I5, I3, I2 and DH270.4, arranged from top to bottom. The bottom-most plot shows histogram of hypervariable V1 loop lengths for autologous viruses that are resistant to all above antibodies. These V1 length distributions across antibodies were statistically compared using a two sided Wilcoxon Rank Sum Test and the bottom panel shows comparisons that yielded p-values < 0.05. **B)** Similar to panel (A) top, except amino acid frequency are shown using logos. To highlight diversity recognition, the amino acids and N-linked glycosylation sites of the consensus form are blanked out of the LOGOs; the consensus form is written along the bottom. The TF form matched the consensus except at position 300, which was Glycine. Variants are color-coded according to which intermediate could first recognize the variant, or if the variants were predominantly found in the resistant virus group. The table below summarizes the variants recognized by each antibody at the variable Env sites.

A

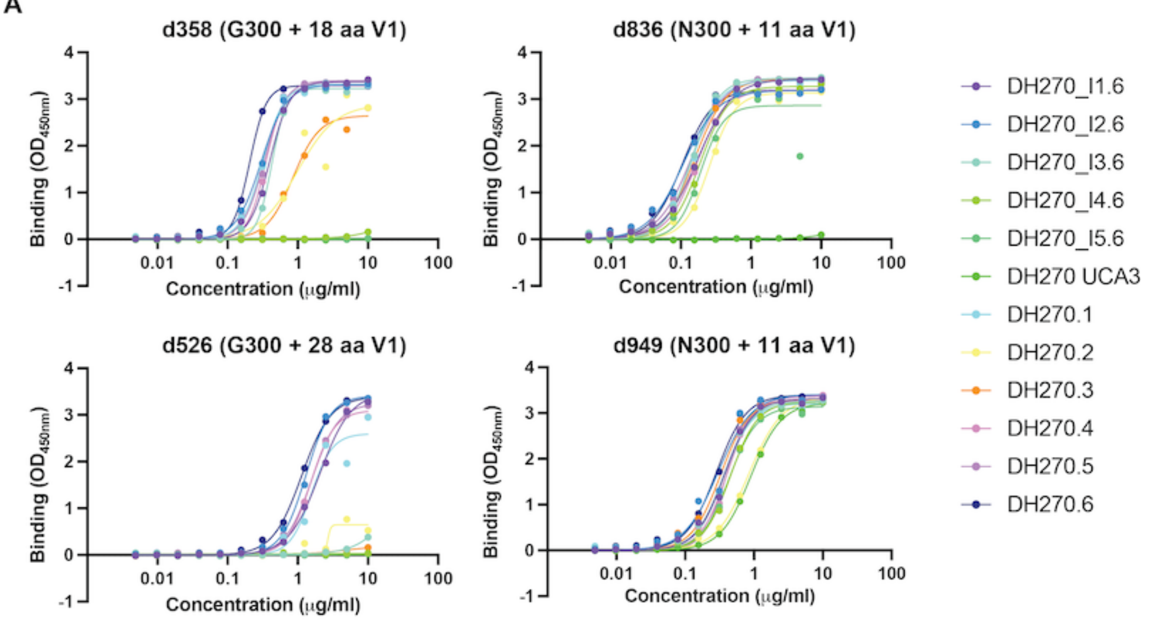

B

	DH270 UCA3	DH270_I5.6	DH270_I3.6	DH270_I2.6	DH270.1	DH270_I1.6	DH270.4	DH270.5	DH270.6
d836	0.00	4.83	6.31	6.20	6.20	5.95	5.60	6.34	6.28
d358	0.00	0.00	4.35	4.96	4.90	4.65	4.74	4.96	5.49
d526	0.00	0.00	0.08	2.97	2.02	2.34	2.49	2.53	3.15
d949	3.28	4.18	4.63	5.13	4.54	4.67	4.64	4.59	5.23

	DH270_I4.6	DH270.2	DH270.3
d836	5.56	4.93	6.13
d358	0.02	2.77	2.69
d526	0.00	0.37	0.04
d949	4.30	3.53	4.88

Log(AUC)	0.00	0.01-1.00	1.01-3.00	3.00-7.00

Supplemental Figure 13. ***Binding of DH270 clonal clone antibodies to CH848 Env SOSIPs measured by ELISA.*** **A)** ELISA binding curves for CH848 d358, d526, d836, and d949. **B)** Table of log area under the curve for ELISA binding curves.

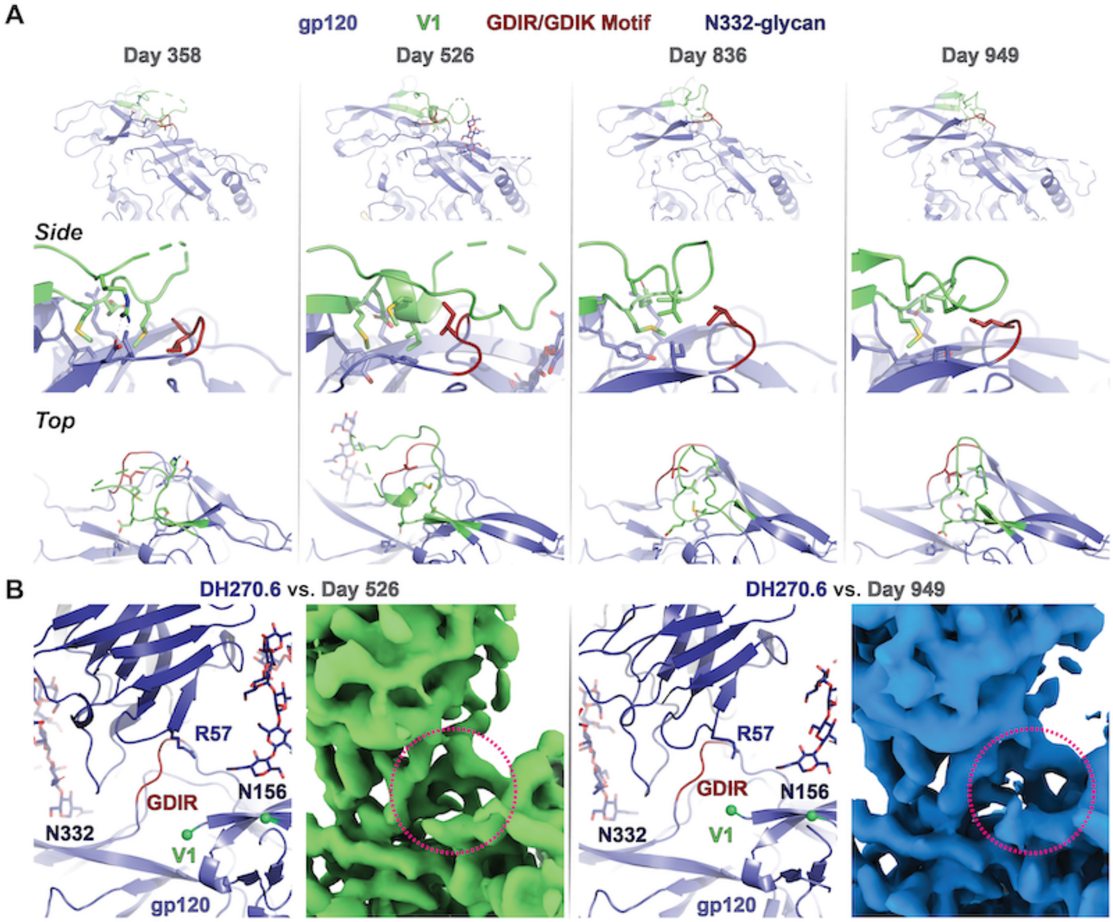

Supplemental Figure 14. Variability in V1 loop length and sequence retains vulnerable Env motif protection. A) Structures of unliganded V3-glycan epitope Env ectodomains for Env sequences isolated from the CH848 infected individual at days 358, 526, 836, and 949 post infection. Upper panels depict a single Env gp120 (blue) protomer with the V1 loop (green) and the V3 GDIR/K motif (red) highlighted. The middle panels present zoomed in, side views of the V3-glycan epitope showing the conserved hydrophobic core residues in stick representation. The lower panel presents a top view of the epitope. **B)** Comparison between the DH270.6 bound d526 and d949 trimer ectodomains showing the fit structure and maps. Green spheres in the structures identify the residue positions at which the gp120 V1 loops are poorly resolved in the maps. The magenta circle over the maps indicates the position of missing gp120 V1 loop densities.

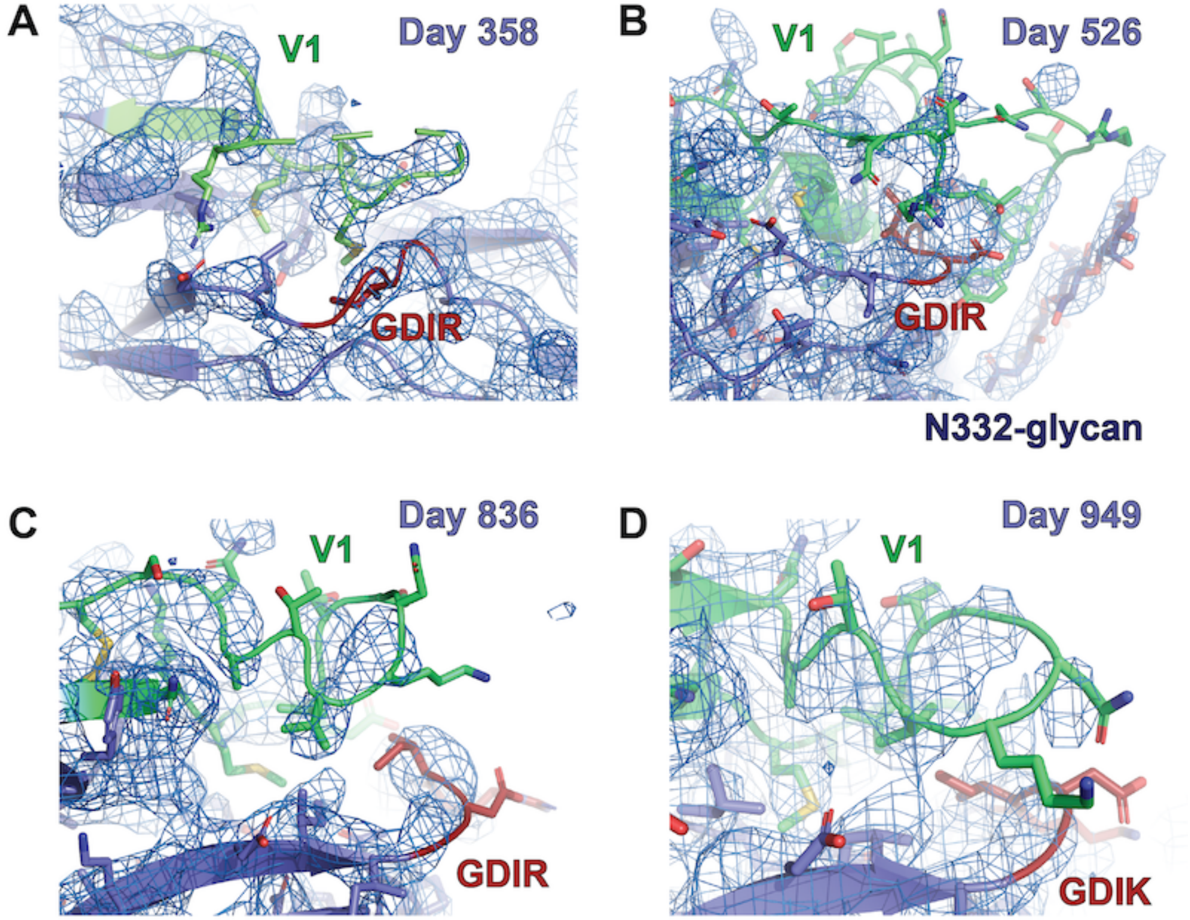

Supplemental Figure 15. ***Unliganded V3-glycan epitopes of CH848 Env with different V1 loop lengths.*** **A-D)** Map and fits of the CH848 day 358, 526, 836, and 949 structures at the V3-glycan epitope.
