# Supplementary material for: Structural basis for breadth development in the HIV-1 V3-glycan targeting DH270 antibody clonal lineage
Source: Nat Commun. 2023 May 15;14:2782. doi: 10.1038/s41467-023-38108-1 (PMC10184639; doi:10.1038/s41467-023-38108-1)
Supplement: Supplementary file 2 — Reporting Summary [file 41467_2023_38108_MOESM2_ESM.pdf]

nature portfolio

Corresponding author(s): Rory Henderson, Barton F. Haynes, Alberto Bartesaghi, Priyamvada Acharya

Last updated by author(s): Feb 8, 2023

Reporting Summary

Nature Portfolio wishes to improve the reproducibility of the work that we publish. This form provides structure for consistency and transparency in reporting. For further information on Nature Portfolio policies, see our Editorial Policies and the Editorial Policy Checklist.

Statistics

For all statistical analyses, confirm that the following items are present in the figure legend, table legend, main text, or Methods section.

n/a	Confirmed	
☐	☒	The exact sample size (*n*) for each experimental group/condition, given as a discrete number and unit of measurement
☒	☐	A statement on whether measurements were taken from distinct samples or whether the same sample was measured repeatedly
☐	☒	The statistical test(s) used AND whether they are one- or two-sided<br>*Only common tests should be described solely by name; describe more complex techniques in the Methods section.*
☒	☐	A description of all covariates tested
☒	☐	A description of any assumptions or corrections, such as tests of normality and adjustment for multiple comparisons
☒	☐	A full description of the statistical parameters including central tendency (e.g. means) or other basic estimates (e.g. regression coefficient) AND variation (e.g. standard deviation) or associated estimates of uncertainty (e.g. confidence intervals)
☐	☒	For null hypothesis testing, the test statistic (e.g. *F*, *t*, *r*) with confidence intervals, effect sizes, degrees of freedom and *P* value noted<br>*Give P values as exact values whenever suitable.*
☒	☐	For Bayesian analysis, information on the choice of priors and Markov chain Monte Carlo settings
☒	☐	For hierarchical and complex designs, identification of the appropriate level for tests and full reporting of outcomes
☒	☐	Estimates of effect sizes (e.g. Cohen's *d*, Pearson's *r*), indicating how they were calculated

Our web collection on statistics for biologists contains articles on many of the points above.

Software and code

Policy information about availability of computer code

Data collection	Latitude 3.51
Data analysis	Pymol 2.5.2, VMD 1.9.4, cryoSPARC v4, Relion 3.1, UNBLUR 1.0, CTFFIND 4.1.14, cisTEM 1.0, UCSF Chimera 1.6, UCSF ChimeraX 1.5, Isolde 1.4, Coot 0.9.8.7, Schrodinger 2022-1, Phenix 1.2, R 4.2.2, Biacore T200 Evaluation Software 3.2

For manuscripts utilizing custom algorithms or software that are central to the research but not yet described in published literature, software must be made available to editors and reviewers. We strongly encourage code deposition in a community repository (e.g. GitHub). See the Nature Portfolio guidelines for submitting code & software for further information.

Data

Policy information about availability of data

All manuscripts must include a data availability statement. This statement should provide the following information, where applicable:

- Accession codes, unique identifiers, or web links for publicly available datasets
- A description of any restrictions on data availability
- For clinical datasets or third party data, please ensure that the statement adheres to our policy

Cryo-EM reconstructions and atomic models generated during this study are available at wwPDB and EMBD (www.rcsb.org; http://emsearch.rutgers.edu) under the following accession codes: EMD IDs: EMD-40282, EMD-40283, EMD-40285, EMD-40286, EMD-40287, EMD-40288, EMD-40291, EMD-40290, EMD-40289, EMD-40284, EMD-40280, EMD-40280, EMD-40278, EMD-40277, EMD-40275, EMD-40281, EMD-40279, EMD-40274, EMD-40273 and PDB IDs: 8SAW, 8SAX, 8SAZ, 8SB0, 8SB1, 8SB2, 8SB5, 8SB4, 8SB3, 8SAY, 8SAU, 8SAU, 8SAS, 8SAR, 8SAQ, 8SAV, 8SAT, 8SAN, 8SAL

Human research participants

Policy information about studies involving human research participants and Sex and Gender in Research.

Reporting on sex and gender	n/a
Population characteristics	n/a
Recruitment	n/a
Ethics oversight	n/a

Note that full information on the approval of the study protocol must also be provided in the manuscript.

Field-specific reporting

Please select the one below that is the best fit for your research. If you are not sure, read the appropriate sections before making your selection.

☒ Life sciences ☐ Behavioural & social sciences ☐ Ecological, evolutionary & environmental sciences

For a reference copy of the document with all sections, see nature.com/documents/nr-reporting-summary-flat.pdf

Life sciences study design

All studies must disclose on these points even when the disclosure is negative.

Sample size	Cryo-EM dataset information is provided in the cryo-EM data tables.
Data exclusions	No data were excluded in this study.
Replication	Each cryo-EM reconstruction was constructed from between 35,000 and 400,000 independent particles. The binding studies were each representative of at least two independent experiments
Randomization	Cryo-EM particles were picked in an automated and unbiased manner. Thus, randomization of the particles that were picked for processing was in-built within the methods used
Blinding	Blinding is not typical or standard in the field of cryo-EM structural biology analysis or in in vitro study of the type presented here.

Reporting for specific materials, systems and methods

We require information from authors about some types of materials, experimental systems and methods used in many studies. Here, indicate whether each material, system or method listed is relevant to your study. If you are not sure if a list item applies to your research, read the appropriate section before selecting a response.

Materials & experimental systems

n/a	Involved in the study
☐	☒ Antibodies
☐	☒ Eukaryotic cell lines
☒	☐ Palaeontology and archaeology
☒	☐ Animals and other organisms
☒	☐ Clinical data
☒	☐ Dual use research of concern

Methods

n/a	Involved in the study
☒	☐ ChIP-seq
☒	☐ Flow cytometry
☒	☐ MRI-based neuroimaging

Antibodies

Antibodies used	DH270 clonal lineage IgG, VRC01 IgG, and PGT151 IgG
Validation	Cloned in-house and verified by DNA sequencing. Purified antibody validated using SDS-PAGE and binding to HIV-1 Env Ectodomain ectodomain by SPR and/or ELISA.

Eukaryotic cell lines

Policy information about cell lines and Sex and Gender in Research

Cell line source(s)	Freestyle 293 and Expi293 cells
Authentication	Manufacturer of the cell line provides authentication information on their website
Mycoplasma contamination	All cell lines tested negative for mycoplasma contamination
Commonly misidentified lines (See ICLAC register)	n/a
